# Hsp90-Mediated Multi-Drug Resistance in DNA Polymerase-Defective Strains of *Candida albicans*

**DOI:** 10.3390/jof10030222

**Published:** 2024-03-19

**Authors:** Bhabasha Gyanadeep Utkalaja, Satya Ranjan Sahu, Sushree Subhashree Parida, Narottam Acharya

**Affiliations:** 1 Laboratory of Genomic Instability and Diseases, Department of Infectious Disease Biology, Institute of Life Sciences, Bhubaneswar 751023, India; 2 Regional Center of Biotechnology, Faridabad 121001, India

**Keywords:** DNA replication, DNA polymerase epsilon, *Candida*, candidiasis, Hsp90, azoles, drug resistance, biofilm

## Abstract

The incidence of infections caused by *Candida* species, specifically by drug-resistant isolates, is a major health concern as they can disseminate to and colonize most vital organs, enhancing morbidity and mortality. Several molecular mechanisms have been reported to be involved in drug resistance. These are mostly drug- and isolate-specific. Here, we characterized three different genetically modified strains of *C. albicans* that were multi-drug-resistant (MDR) and deciphered a uniform mechanism responsible for resistance. DNA polymerase epsilon (Polε) is a leading strand-specific polymerase consisting of four subunits, namely, Pol2, Dpb2, Dpb3, and Dpb4. The deletion of one or both of the Dpb3 and Dpb4 subunits in *C. albicans* rendered multi-drug resistance. A detailed characterization of these strains revealed that acquired mutagenesis, drug efflux pumps, and other known mechanisms did not play a significant role because the complemented strain showed drug sensitivity. More importantly, the function of heat shock protein 90 (Hsp90) in these knockout strains is critical for reducing susceptibility to several antifungal drugs. Cell wall deformity and composition in these strains can add to such a phenotype. The inhibition of Hsp90 function by geldanamycin and tricostatin A sensitized the MDR strains to antifungals. Considering our earlier research and this report, we suggest that replication stress induces Hsp90 expression and activity in order to orchestrate a cellular stress response circuit and thus develop fungal drug resistance. Thus, Hsp90 is an important drug target for use in combinatorial therapy.

## 1. Introduction

*Candida albicans* is the most frequently isolated clinical species, being responsible for the majority of candidiasis burden in the USA and Europe, followed by other non-albicans species like *C. glabrata*, *C. parapsilosis*, *C. tropicalis*, *C. dubliniensis*, *C. krusei*, *C. lusitaniae*, and *C. auris* [1,2]. Although *Candida* species are commensals and evolve normally as an integral part of the healthy human microbiome, they can cause infections ranging superficial mucosal to disseminated candidiasis in immune-suppressed individuals [3]. Since *Candida* develops a biofilm on medical devices, nosocomial candidiasis is quite common [4]. About 1.5 million lethal fungal infections are reported each year, which is the same as the number of deaths caused by tuberculosis or HIV, and more than malaria or breast or prostate cancer [5]. Currently, four main classes of antifungal drugs, including azoles, polyenes, echinocandins, and 5-Flurocytosine (5FC), are being recommended [6]. The widespread and frequent use of these drugs, specifically azoles, has led to the rapid evolution of antifungal drug resistance in *Candida* species. Moreover, the lack of availability of effective vaccines against any fungal pathogens for human use makes fungal infections very serious and turns them into a constant threat to public health worldwide [2]. Unsurprisingly, the WHO has recently listed several *Candida* species including *C. albicans* as priority pathogens, advocating for improved diagnostics, the monitoring of antifungal resistance, and research and innovation to develop effective therapeutics [7].

Several molecular mechanisms have been suggested for developing *C. albicans* antifungal drug resistance, and targeting those mechanisms is one of the priority areas in efforts to improve the efficacy of the existing range of antifungal drugs [6,8,9,10]. Azole causes the depletion of ergosterol in the membrane and the accumulation of toxic sterol and reactive oxygen species (ROS) in the fungal cells. The combination of these events suppresses fungal growth. In *C. albicans*, the *ERG11* and *ERG3* genes encode for lanosterol demethylase and C-5 sterol desaturase of the ergosterol biosynthetic pathway, respectively. Amino acid substitution(s), responsible for the non-functionality of *ERG11* and *ERG3*, results in drug resistance [11,12]. Upc2, a transcription factor, regulates the activities of ergosterol biosynthetic genes, and several mutations in CaUpc2 have been identified in azole-resistant clinical isolates [13]. The overexpression of two drug efflux systems’ ATP-binding cassette (ABC) families of *C. albicans* drug resistance (CDR) transporters, e.g., Cdr1p and Cdr2p, and major facilitator superfamily (MFS) transporters, e.g., Mdr1, are frequently observed in the clinical isolates of *C. albicans* [14,15]. While the Tac1 transcription factor regulates the expression of *CDR1* and *CDR2*, *MDR1* is regulated by Mrr1. Certain mutations in *TAC1* and *MRR1*, causing constitutive overexpression of their respective drug transporters, were found to induce azole resistance [16,17]. Trisomy correlates with increased azole resistance in most of the chromosomes of *C. albicans* [18]. For example, chromosome 3 possesses *CDR1* and *CDR2*, and chromosome 6 carries *MDR1*, and an increase in the copy number of these chromosomes leads to the overexpression of respective transporters and azole resistance [19]. Polyenes like amphotericin B (Amp B) and nystatin bind to ergosterol and depolarize the membrane to cause cell death [6,20]. Although polyene resistance in clinical isolates is relatively less explored, few reports link it to *ERG3* and *ERG6* functions in *C. albicans* and *C. glabrata* [21]. Echinocandins, including caspofungin, micafungin, and anidulafungin, target the β1-3 glucan synthase enzyme, a complex of Fks1, Fks2, and Fks3 proteins [22,23]. Mutations in *CaFKS1* are observed in echinocandin-resistant clinical isolates. Echinocandins alter the composition and stability of the cell wall and induce stress in the cell. As a counteractive response, the fungal cell activates a series of signaling cascades that involve protein kinase C (PKC), calcineurin, and Hsp90 to protect the cell against such destabilization [24]. 5-Flurocytosine (5FC) is a nucleic acid biosynthesis inhibitor. Cytosine permease, which imports 5FC, and cytosine deaminase, which metabolizes it into 5-Flurouracil (5FU), are two fungal-specific enzymes. The mutational inactivation of these enzymes causes increased resistance to 5FC [18].

Defects in DNA polymerase function increase mutagenesis and genome instability in cells [25]. Thus, acquired mutations in DNA polymerase-defective *C. albicans* strains can induce MDR. In eukaryotes, DNA replication is coordinated by three essential DNA polymerases, namely Polα, Polδ, and Polε [25]. Extensive genetic and biochemical analyses in *Saccharomyces cerevisiae* suggested that Polε is only involved in leading strand DNA synthesis, whereas Polδ synthesizes both leading and lagging strands of DNA [26]. Polα-primase provides the RNA–DNA primer required to initiate DNA replication from the origins. In *S. cerevisiae* and *C. albicans*, the Polδ holoenzyme consists of Pol3, Pol31, and Pol32 subunits [27]. Pol32 subunit is dispensable in both the yeasts; however, in its absence, the processivity and fidelity of Polδ become compromised and *C. albicans* cells exhibit a slow growth phenotype; sensitivity to DNA-damaging agents; an increased rate of heterozygosity loss; the accumulation of genome instability, mostly in the intergenic and repetitive sequence regions of the genome; and resistance to only azole drugs [27]. Surprisingly, the azole drug resistance phenotype was not due to acquired mutations that accumulated in *pol32*ΔΔ, but rather due to Hsp90’s function and cell wall/membrane deformity [27]. In this study, we explored the drug resistance mechanism in Polε-defective strains of *C. albicans*. Polε consists of four subunits: Pol2 is the catalytic protein and Dpb2, Dpb3, and Dpb4 are the accessory proteins. Unlike the *pol32*-defective strain, both single and double-gene knockout strains (*dpb3*ΔΔ, *dpb4*ΔΔ, and *dpb3*ΔΔ*dpb4*ΔΔ) exhibited similar resistance phenotypes to a wide range of antifungal drugs. The complemented strain showed sensitivity and ruled out any major contribution of genome instability to drug resistance. After ruling out several mechanisms of drug resistance, we confirmed that the expression of Hsp90 increases under genomic stress and its activity is required for drug resistance. Thus, we concluded that Hsp90 plays a universal role in anti-fungal drug resistance in DNA polymerase-defective strains of *C. albicans*, is induced upon replication stress, and can be explored as a drug target to tackle drug-resistant isolates.

## 2. Materials and Methods

### 2.1. Reagents, Strains, and Growth Conditions

The oligonucleotides used in this study were procured from Eurofins Scientific, United States of America (USA), and Integrated DNA Technologies, USA. Antifungal drugs like fluconazole, ketoconazole, miconazole, amphotericin B, berberine, CFW, CsA, CaCl_2_, SDS, Congo red, aniline blue, and DCFH-DA were obtained from Sigma Aldrich, India. 5FU, 5FC, trichostatin A, and geldanamycin were purchased from TCI, India, and caspofugin was procured from Glenmark Pharmaceuticals, India. SYTOX Green and concanavalin A were obtained from Thermo Fisher Scientific, USA. Fetal bovine serum of South American origin was purchased from PAN Biotech, GmbH, Germany. Wild-type C. albicans SC5314 and its derivative strains were grown in liquid and agar containing yeast extract peptone dextrose (YPD) media without and with various drugs at 30 °C, as per the requirements.

### 2.2. Growth Curve Assay

Overnight-grown cultures of various knockout strains of *C. albicans* were diluted with YPD broth to achieve OD_600_ nm = 0.1 in 10 mL of total volume. The cultures were allowed to grow at 30 °C in 200 RPM shaking conditions, without or with the mentioned dose of antifungal compounds. Absorbance was measured at an interval of 2–3 h for 16–30 h. Experiments were carried out twice with biological duplicates. The growth curve was plotted using GraphPad Prism 8.0 software by taking the OD values of each strain.

### 2.3. Antifungal Drug Susceptibility Assay by Spot Dilution Method

Drug sensitivity assay was described as before [28]. Briefly, the overnight pre-cultures of *C. albicans* were diluted to an OD_600_ = 1 in YPD media. The samples were further serially diluted 10-fold in a 96-well round-bottom plate and spotted using a spotter onto YPD + agar plates, without or with different concentrations of drugs like fluconazole (3.7 μM, 7.5 μM, 11.25 μM), ketoconazole (0.5 μM, 0.75 μM, 1 μM), miconazole (0.8 μM, 1.2 μM, 1.6 μM), 5Flurocytosine (40 μM, 80 μM, 160 μM), 5Fluorouracil (80 μM, 160 μM, 240 μM), amphotericin B (71.4 nM, 107 nM, 142.8 nM), caspofungin (32 μM, 48 μM, 64 μM), berberine (48 μM, 96 μM, 144 μM), CsA (1 μM, 2 μM, and 3 μM), SDS (0.04%, 0.05%, and 0.06%), CFW (1 μM, 1.5 μM, 2 μM), CaCl_2_ (350 mM, 500 mM, and 750 mM), rapamycin (1.2 ng/mL, 2.56 ng/mL, 3.8 ng/mL), Congo red (0.01%, 0.015%, 0.02%), geldanamycin (10 µM and 20 µM), and trichostatin A (0.8 µM and 1.6 µM). To inhibit the Hsp90 function, a similar sensitivity test was conducted with a slight modification. Plates were prepared in combination with antifungal drug fluconazole (6 μM) or amphotericin B (107 nM) using geldanamycin (10 µM and 20 µM) or trichostatin A (0.8 µM and 1.6 µM). The spotted plates were further placed inside a 30 °C incubator for 48 h and imaged using the Chemi XRS Gel Documentation system (Bio-Rad, USA).

### 2.4. CFU Analysis

The colony-forming unit assay was described as before [28]. For a quantitative survival experiment in response to rapamycin, logarithmically growing cells of WT and *dpb3*ΔΔ*dpb4*ΔΔ were diluted to ~500 cells/mL in YPD broth, from which 200 μL was spread onto YPD plates, with or without containing different concentrations of rapamycin (1–4 ng/mL). After 2–3 days of incubation, the number of colonies on each plate was counted and plotted using GraphPad Prism 8.0 software. Experiments were carried out twice with biological duplicates.

### 2.5. Membrane Permeability Assay

In order to determine the membrane permeability of WT and *dpb3*ΔΔ*dpb4*ΔΔ in the presence of fluconazole, 2 × 10^6^ cells from overnight-grown cultures were taken up in a 1.5 mL microcentrifuge tube. The cell pellet was washed thrice with 1 x PBS and resuspended in 1 mL of the same buffer. The cells were treated without and with fluconazole (3.7 μM) and incubated further at 30 °C in a shaker for 8 h. Cells were pellet down at 12,000 rpm for 1 min, washed twice, and resuspended in 1 mL PBS. SYTOX Green staining was performed with a final concentration of 1 μg/mL and the products were incubated for 30 min in a 30 °C in the dark. Again, cells were washed with and resuspended in 500 µL PBS. Stained cells were observed under a fluorescence microscope. Cells were acquired in a BD LSR Fortessa Flow cytometer via blue laser excitation (488 nm). Data were analyzed in Flowjo software 8.2.0 and an average of three data sets were plotted. The analyzed data were exported in the JPEG format.

### 2.6. Measurement of ROS

Intracellular ROS production was estimated similarly to the membrane permeability assay, except that in place of SYTOX Green, 10 μM of DCFH-DA dye was used. Stained samples were washed twice with PBS followed by resuspension in 1 mL PBS. The DCFH- (DA positive cells were detected via flow cytometry (excitation:emission wavelength::485:530 nm). Data were analyzed in Flowjo software and an average of three data sets was plotted. Analyzed data were exported in the JPEG format.

### 2.7. Gene Expression Analysis by RT-PCR

To determine the mRNA expression of different genes related to drug resistance in *C. albicans*, total RNA was isolated from overnight-grown cultures of WT and *dpb3*ΔΔ*dpb4*ΔΔ strain using the MagSure^TM^ all RNA Isolation kit (RNA Biotech, India, #MAR-100). RNA was quantified using a NanoDrop 2000 (Eppendorf, Germany) and absorbance was recorded at 260/280 and 260/230 ratios. About 2 µg of RNA was used to synthesize cDNA using a high-capacity cDNA reverse transcription kit from Invitrogen with the provided random primers. A total volume of 20 µL of qRT PCR reaction mixture was set up, containing 100 ng of cDNA, 10 pmol of forward and reverse primers, and 2 x SYBR green qPCR Master mix (Applied biosystem, Cat#A25742). The qRT PCR cycling was carried out in Quant Studio 3 with fast cycling conditions for 2 min, including 95 °C, followed by 40 cycles of 95 °C for 5 s for denaturation and 60 °C for 30 s for annealing and extension. All the experiments were performed in biological duplicates with technical triplicates. The data obtained were analyzed using the 2^−ΔΔCT^ method. The gene expression of *CDR1*, *CDR2*, *MDR1*, *HSP30*, *HSP90*, *ERG11*, and *ERG3* was analyzed by normalizing with *GAPDH* as the housekeeping gene, and the fold changes with respect to the control were plotted using GraphPad Prism 8 software. Semi-quantitative RT PCR of the above-mentioned genes was also performed in a 20 µL reaction with 100 ng of cDNA, 10 pmol primer mix for each gene, 200 μM of dNTPs, 1X Taq buffer, and 1U of Taq DNA polymerase (Sigma). The PCR conditions used were an initial denaturation at 95 °C for 1 min, followed by 30 cycles of 95 °C for 30 s, 60 °C for 30 s and 72 °C for 30 s. The amplified products were resolved in 1% agarose gel and images were captured using a Chemi XRS Gel Documentation system (Bio-Rad, USA). The primer sequences of various genes used for real-time PCR are given in Table 1.

### 2.8. Western Blot Analysis

About 1 mL of overnight-grown cultures of WT and *dpb3*ΔΔ*dpb4*ΔΔ strains was harvested and the pellets were resuspended in 200 μL of lysing buffer (50 mM Tris HCl pH 7.5, 150 mM NaCl, 1 mM EDTA, NP40 1%, 1 mM PMSF), supplemented with 1 x protease inhibitor cocktail (Biopioneer). Cell lysates were electrophoresed in a 10% SDS-PAGE gel and the extract was transferred to a 0.45 μm PVDF membrane by applying 90 V for 2 h. Blocking was carried out with 5% BSA diluted in 1 x TBST for 2 h. The blot was again gently rinsed once to remove excess BSA and then incubated with a primary antibody of HSP90 (CST #4874) with a dilution of 1:1000 with 1 x TBST overnight. After that, blots were washed thrice with 1 x TBST for 5 min each. The secondary anti-rabbit antibody (cat no: CST-7074S, 1:5000) conjugated to HRP was added and incubated for 1 h at room temperature. After incubation, blots were again washed five times for 5 min each. Bands were visualized with WesternBLoT Chemiluminescence HRP substrate ECL (TaKaRa) on the ChemiDoc Imaging System (Biorad, USA).

### 2.9. Transmission Electron Microscopy

The ultrastructures of WT and *dpb3*ΔΔ*dpb4*ΔΔ *C. albicans* cells were examined via TEM using a protocol described before [27]. After the fixation and hardening of the samples, they were sectioned using Leica EM UC7 microtome. The sections were collected on the copper grid and stained with uranyl acetate for 30 min. This was followed by 3 washing times with distilled water, and the sections were allowed to dry for 2 h. Samples on the grid were visualized under the JEM-2100Plus JEOL TEM imaging machine (JEOL, Japan) and images with different magnifications were taken at different focal places. The thickness of the cell wall was measured by using Image J software V 1.5.3.

### 2.10. Cell Wall Components Estimation

The overnight-grown cultures of various strains of *C. albicans* were diluted to OD_600_ to 0.5. About 1 mL of the diluted sample was centrifuged for 1 min at 12,000 rpm. The pellet was washed thrice and resuspended in 1 mL PBS. To estimate the chitin content, 2.5 μg/mL CFW was added to the sample. For β1,3-glucan staining, aniline blue (2.5%) was used and 1 mg/mL Con A tetramethyl rhodamine was added for mannan estimation. Staining was carried out for 30 min at 30 °C in the dark. After incubation, cells were again washed with PBS, resuspended in 500 μL PBS, and transferred to FACS tubes for flow cytometry in an LSR Fortessa^TM^ cell analyzer. For CFW and aniline blue, a UV laser was used with an excitation wavelength (350 nm) and a bandpass filter (450/50 nm). For Con A, a blue green laser was used that had an excitation wavelength (561 nm) with a bandpass filter (568/15 nm). The experiment was carried out thrice and an average of mean florescence intensity was determined.

### 2.11. Berberine Accumulation Assay

*C. albicans* cells from overnight-grown cultures were harvested and washed twice with 1 x PBS and resuspended in PBS to obtain approximately 5 × 10^7^ cells/mL. About 10 μg/mL concentration of berberine was added to each sample and this was incubated at 30 °C in a 200 rpm shaking condition. A total of 1 mL of mixed sample was collected at intervals of 10 min for 2 hr. Cells were harvested via centrifugation for 1 min at 12,000 rpm and the supernatant was removed carefully. Pellets were washed twice and resuspended in 1 mL of PBS. A total of 150 μL samples were aliquoted in a black 96-well microplate with a clear bottom for fluorescence measurement in the ELISA plate reader. The excitation and emission wavelengths were 360 nm and 520 nm, respectively.

### 2.12. Biofilm Assay

The biofilm assay was carried out as described previously [29]. Briefly, the overnight-grown fungal cultures were diluted up to an OD600 of 0.5 in YPD nutrient media. About 10 µL of diluted culture was grown in 990 µL YPD media without and with 10% serum in 24-well polystyrene plates (Cat #3527, Corning) for 24 h at 30 °C. The supernatant was carefully pipetted out without disturbing the mature biofilm at the base of the well. The plate was placed in an inverted position on a tissue bed to discard any remaining media with planktonic cells without disturbing the resultant biofilm. The plate was washed two times gently with 1 mL of 1 x PBS. The biofilm was then treated with 500 µL of 0.1% crystal violet stain for 20–30 min at room temperature, followed by a wash with distilled water, and allowed to dry at room temperature for 5 h. Images of the stained plate were taken using a Biorad molecular imager Gel DocTM XR+ imaging system. The bound dye was then resuspended in 1 mL of 33% glacial acetic acid and incubated further for 1 h at room temperature. The experiment was conducted in duplicate. The absorbance was recorded at 570 nm in an ELISA plate reader (Perkin Elmer, USA).

### 2.13. Biofilm Detection by CLSM

Various strains of *C. albicans* were grown overnight and diluted to an OD_600_ of 0.5. Some 10 μL of diluted culture was mixed with 990 μL of fresh YPD liquid media and transferred to the 6-well cell culture plate (Cat# 3516, Corning) containing glass cover slips and allowed to grow at 30 °C for 24 h. After 24 h, the supernatant was carefully removed with a pipette, followed by washing with 500 µL of 1 x PBS. For staining, 20 μL of 1% acridine orange was added and incubated for 20 min in dark. Excess staining was removed by washing with 1 x PBS, followed by cell fixation with 200 μL of 4% formaldehyde for 30 min at room temperature. To remove excess fixatives, washing was performed three times with 1X PBS. Finally, the glass coverslips were placed gently onto the glass slide, and images were captured in the Leica TCS SP8 confocal scanning system with an excitation wavelength of 483 nm and in a bandpass filter with an emission wavelength ranging from 500 to 510 nm.

### 2.14. Statistical Analysis

The statistical analysis of data sets derived from the growth curve, cell permeability, ROS production, RT-PCR, cell wall component measurement, etc., assays was carried out using GraphPad Prism 8.0 based on a two-way ANOVA multiple comparison test. The level of significance was determined, and stars were given in graphs based on *p*-values (* *p* ≤ 0.05, ** *p* ≤ 0.01, *** *p* ≤ 0.001, and **** *p* ≤ 0.0001).

## 3. Results

### 3.1. Absence of Accessory Subunits of Polε Induces Azole Drug Resistance in C. albicans

**Figure 1:**
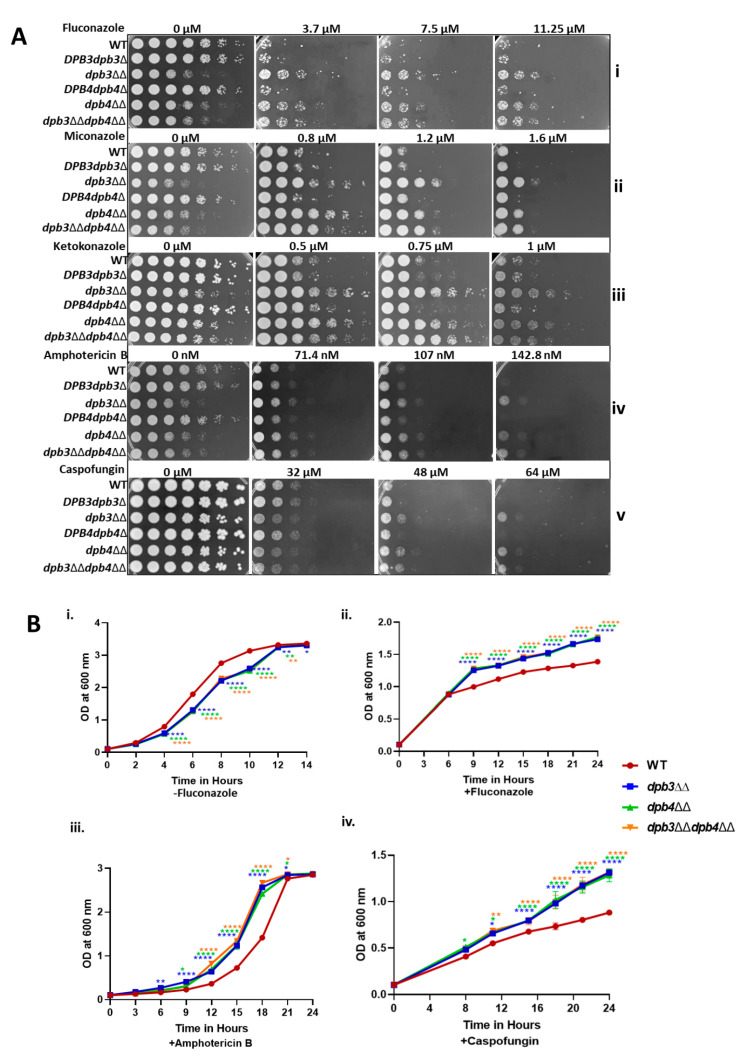
Effect of antifungal drugs on *C. albicans* strains. (**A**) Overnight cultures of WT, *DPB3dpb3*Δ, *dpb3*ΔΔ, *DPB4dpb4*Δ, *dpb4*ΔΔ, and *dpb3*ΔΔ*dpb4*ΔΔ strains were serially diluted and spotted onto YPD plates, with or without the indicated concentrations of azoles (**i**–**iii**), Amp B (**iv**), and caspofungin (**v**) drugs. All the plates were incubated at 30 °C for 48 h and photographed. (**B**) Overnight-grown cultures of WT, *dpb3*ΔΔ, *dpb4*ΔΔ, and *dpb3*ΔΔ*dpb4*ΔΔ strains were diluted in fresh YPD media and grown at 30 °C in the absence (**i**) or presence of FLC (6 μM, (**ii**)), Amp B (75 nM, (**iii**)), and caspofungin (40 μM, (**iv**)). The absorbance was measured at OD_600_ till 14 h for untreated and 24 h for treated versions at regular intervals, and the average two experiments with two biological replicates were plotted. The statistically significant differences (* *p* ≤ 0.05, ** *p* ≤ 0.01, and **** *p* ≤ 0.0001) between the results of WT and mutant strains were determined by using a two-way ANOVA test (Dunnett’s multiple comparisons).

**Figure 2:**
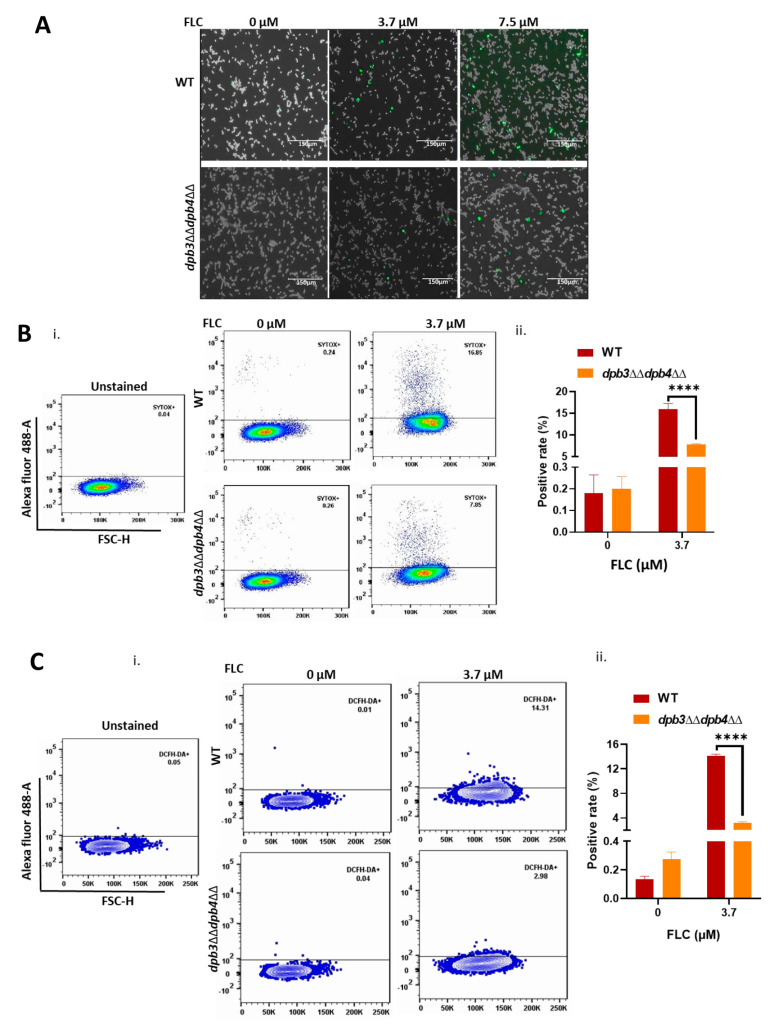
Effect of fluconazole on cell membrane permeability and ROS production in *C. albicans* strains. (**A**) Membrane permeability of WT and *dpb3*ΔΔ*dpb4*ΔΔ strains in response to FLC was determined using SYTOX Green dye and analyzed via microscopy. (**B**) A similar SYTOX green staining assay was carried out, analyzed (**i**), and measured via flow cytometry (**ii**). (**C**) Intracellular ROS accumulation in WT and *dpb3*ΔΔ*dpb4*ΔΔ strains due to FLC treatment was determined by using DCFH-DA dye and analyzed (**i**) and estimated via flow cytometry (**ii**). Analysis of unstained cells as a control was also performed. Asterisks indicate (**** *p* < 0.0001) the statistically significant differences in various results between WT and mutant strains, as determined by using a two-way ANOVA test (Sidak’s multiple comparisons). No star suggests that there was no statistical difference between the data.

Imidazole and triazole are two classes of azole drugs that vary in their structures due to the presence of a number of nitrogens in their azole rings. The imidazole group, which includes econazole, clotrimazole, miconazole, ketoconazole, and tioconazole, possesses two nitrogens, whereas the triazoles, e.g., fluconazole (FLC), voriconazole, posaconazole, and itraconazole, contain three nitrogens in the azole ring. These drugs effectively work against superficial mucosal fungal infection caused by the *Candida* species as well as other fungal pathogens [6,30]. We took advantage of readily available various Polε knockout strains of *C. albicans*, such as *DPB3dpb3*Δ, *dpb3*ΔΔ, *DPB4dpb4*Δ, *dpb4*ΔΔ, and *dpb3*ΔΔ*dpb4*ΔΔ, and determined their susceptibility to azole drugs (Figure 1A). Although the Dpb3 and Dpb4 subunits are non-essential for survival in yeast, they play important roles in stabilizing the Polε holoenzyme structure [25,31]; therefore, deletion strains of *C. albicans* (*dpb3*ΔΔ, *dpb4*ΔΔ, and *dpb3*ΔΔ*dpb4*ΔΔ) showed slow growth phenotype, as evident from both spot and growth curve analyses (Figure 1A,B(i)). Despite the occurrence of growth defects at normal physiological conditions, all the homozygous deletion strains were surprisingly resistant to all three azole drugs, while the heterozygous deletion strains *DPB3dpb3*Δ and *DPB4dpb4*Δ and the isogenic wild-type versions were sensitive and grew poorly, as depicted in the spot assay (Figure 1A(i–iii)). Even in the liquid growth curve assay, the *dpb3*ΔΔ, *dpb4*ΔΔ, and *dpb3*ΔΔ*dpb4*ΔΔ strains exhibited azole resistance phenotypes (Figure 1B(ii), Supplementary Table S1a,b). The double-deletion strain *dpb3*ΔΔ*dpb4*ΔΔ exhibited a similar level of resistance to azoles as the individual subunit deletion, which again suggested that both the genes are epistatic and critically required in the same genetic pathway. Since azoles inhibit ergosterol biosynthesis and alter its concentration in the cell membrane, resulting in increased permeability, we compared the leaky membrane status of the drug-resistant cells with wild-type strains via staining with SYTOX Green dye. We performed analysis via fluorescence microscopy and flow cytometry. SYTOX Green binds to DNA; thus, increased binding to DNA indicates the higher permeability of the cell and nuclear membrane. Roughly equal numbers of wild-type and *dpb3*ΔΔ*dpb4*ΔΔ *C. albicans* cells were first exposed to a susceptible concentration of FLC for 8 h at 30 °C and then stained with SYTOX Green dye for 30 min and analyzed. We observed that, upon treatment with fluconazole, nearly a 2.5-fold greater population of wild-type strains was stained with the dye in comparison to *dpb3*ΔΔ*dpb4*ΔΔ azole-resistant cells (Figure 2A,B(i,ii)). In addition to inhibiting ergosterol biosynthesis, azoles are also known to increase reactive oxygen species (ROS) levels in fungal cells [32]. Next, we measured the endogenous level of ROS in the Polε-defective strains via a real-time fluorogenic assay, using 2′,7′-dichlorofluorescin diacetate (DCFH-DA) as a substrate. Similar to our earlier experiment, an equal number of cells was subjected to FLC and DCFH-DA staining. In the presence of ROS, the oxidation of DCFH-DA converts it into DCF, a green fluorescence molecular probe, which can be analyzed via flow cytometry. Our flow cytometry analyses revealed a higher percentage of cells emitting fluorescence in wild-type *C. albicans* strains, suggesting a high level of ROS production in the presence of FLC (~3 folds), whereas *dpb3*ΔΔ*dpb4*ΔΔ cells produced a very low level of ROS, implying resistance to azoles (Figure 2C(i,ii). Altogether, by analyzing three different knockout strains, our results suggested that the loss of any and both of the non-essential subunits of Polε rendered the same azole drug-resistant phenotype. Such a phenotype is most likely due to compromised cell membrane permeability and a decreased level of cellular ROS production upon azole treatment.

### 3.2. Loss of Dpb3 and Dpb4 Subunits of Polε Also Induces Non-Azole Drug Resistance in C. albicans

**Figure 3:**
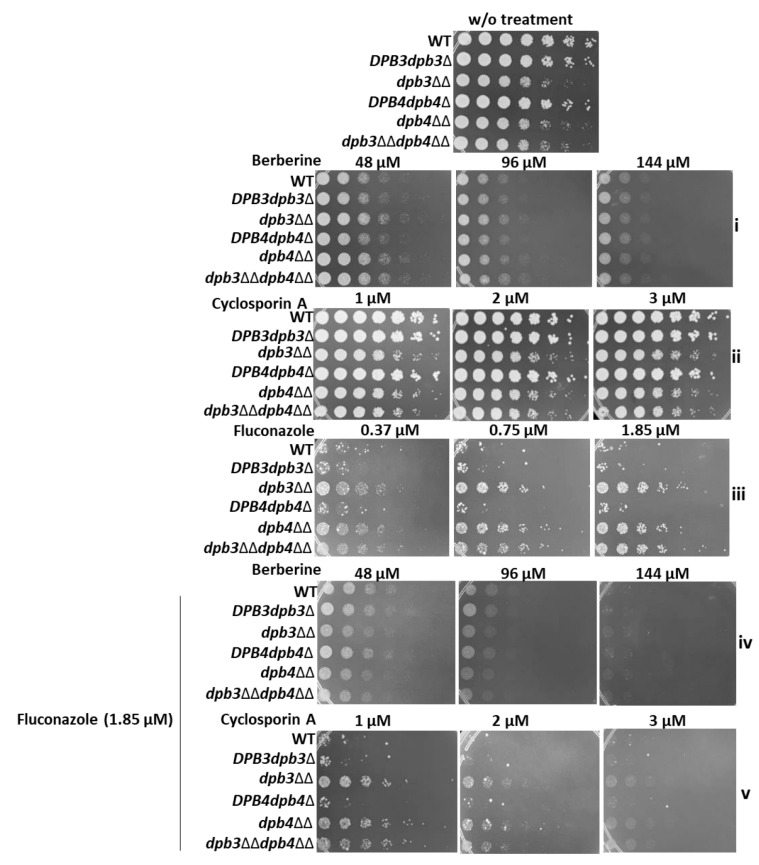
Berberine and Cyclosporin A sensitize fungal cells to fluconazole. A combinatorial effect of berberine (**iv**) and CsA (**v**) with FLC on *C. albicans* cells including the controls (**i**–**iii**) was shown by spotting the cells onto varying concentrations of one drug and keeping the others fixed. All the plates were incubated at 30 °C for 48 h and photographed.

Next, we examined the susceptibility of Polε-defective strains to non-azole drugs like amphotericin B (Amp B), 5FC, 5FU, caspofungin, berberine, and cyclosporine A. Amp B is commonly used to treat aspergillosis, candidiasis, and cryptococcosis, and resistance to Amp B by *Candida* species is rarely found in clinical settings. 5FC is the only pyrimidine analog with antimycotic properties that gets converted to 5FU by a fungal-specific enzyme cytosine deaminase, which is absent in humans. 5FU interferes with fungal DNA, RNA, and protein synthesis. Thus, although 5FC is effective and the safest antimycotic in the healthcare system, it is rarely used in monotherapy due to the rapid development of resistance towards this drug [18]. Caspofungin is the first echinocandin approved for the treatment of a wide range of yeast infections. Berberine is an alkaloid isolated from natural herbs and it has been reported to have antifungal activity [33]. Cyclosporin A (CsA) is isolated from the fungus *Tolypocladium inflatum* and possesses a narrow spectrum of antifungal activity [34]. Both berberine and CsA are two clinically irrelevant drugs, reported to reduce fungal growth in the presence of FLC. Similar to azole sensitivity tests, homozygous deletion strains *dpb3*ΔΔ, *dpb4*ΔΔ, and *dpb3*ΔΔ*dpb4*ΔΔ grew better than the wild-type and heterozygous deletion strains in the presence of Amp B and caspofungin (Figure 1A(iv,v). The growth curve assays also validated the resistance attributes of *dpb3*- and *dpb4*-defective strains (Figure 1B(iii,iv), Supplementary Table S1c,d). We did not observe any significant difference in the susceptibility of wild-type and Polε subunit knockout strains to 5FC, 5FU, berberine, and CsA in the spot assay (Supplementary Figure S1A and Figure 3i,ii). However, the liquid growth curve assays repeatedly showed the mild resistance of Polε-defective strains to 5FC (Supplementary Figure S1B). Since a strain with resistance to a specific drug shows a lesser cellular accumulation of that particular compound, we took advantage of the autofluorescence properties of berberine and carried out a drug accumulation test (Supplementary Figure S1C). As expected, azole-resistant strains accumulated relatively less berberine than the sensitive wild-type *C. albicans* strain. Next, we tested whether berberine and CsA can sensitize Polε-defective strains to azole. To verify this, cells were spotted onto YPD plates containing varying concentrations of berberine or CsA but one constant dose of FLC, and they were compared with individual drug treatments (Figure 3). The FLC-resistant cells grew significantly well on a plate with 1.85 µM of FLC (panel Figure 3iii), exhibited sensitivity to the presence of berberine and CsA (compare panel Figure 3iii with Figure 3iv,v). Altogether, our results confirmed that *dpb3*- and *dpb4*-defective strains are multi-drug-resistant, and the cellular accumulation of antifungals is most likely relatively low in these strains.

### 3.3. Acquired-Mutagenesis-Independent Drug Resistance Mechanism in Polε-Defective Strain of C. albicans

**Figure 4:**
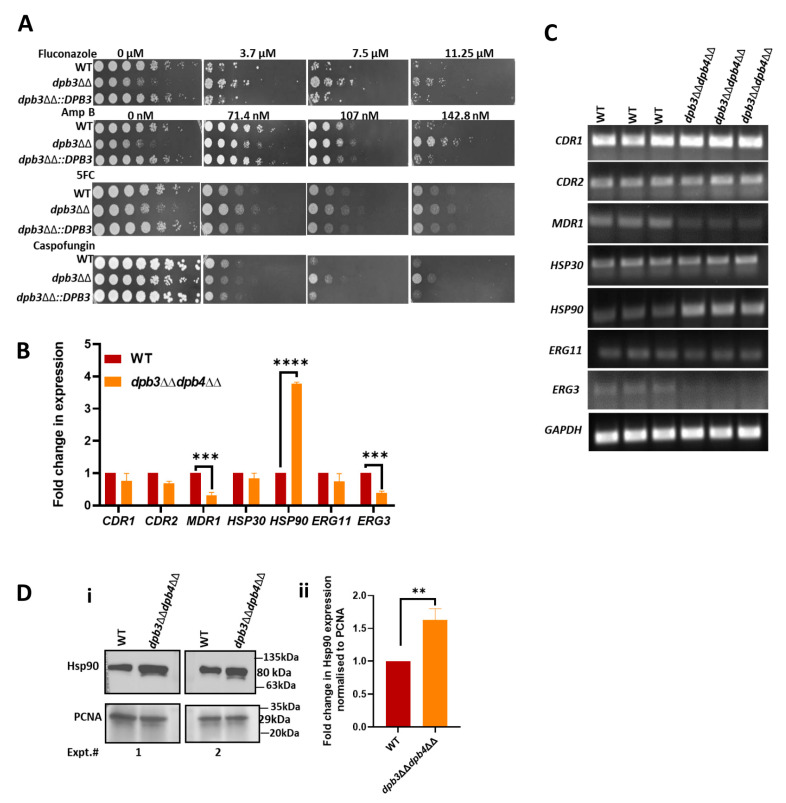
Overexpression of Hsp90 in Polε-defective strain. (**A**) Serially diluted cultures of WT, *dpb3*ΔΔ, and *dpb3*ΔΔ::*DPB3* strains were spotted onto YPD plates without or with indicated concentrations of antifungal drugs. Plates were incubated at 30 °C for 48 h and photographed. (**B**) The expression of genes associated with drug resistance was analyzed using SYBR green in real-time PCR. The fold change between the WT and mutant strain was calculated after determining the 2^−ΔΔCT^ of each gene with respect to *GAPDH* as a housekeeping gene. (**C**) Semi-quantitative RT-PCR was carried out for the mentioned genes and analyzed in an agarose gel electrophoresis. *GAPDH* was used as an internal control. (**D**) The protein level of Hsp90 was checked in WT and *dpb3*ΔΔ *dpb4*ΔΔ via Western analysis by probing with an anti-Hsp90 antibody (**i**) and quantified by using Image J V 1.8.0 (**ii**). The results are presented as mean ± standard deviation. Asterisks indicate (** *p* ≤ 0.01, *** *p* ≤ 0.001, and **** *p* ≤ 0.0001) the statistically significant differences in the mutant strain compared to the results of the WT using a two-way ANOVA test (Sidak’s multiple comparisons).

The absence of *DPB3* and *DPB4* subunits of Polε is known to increase mutagenesis in yeast [35,36]. Acquired mutations in certain genes, especially those involved in drug export and import, drug target genes, cell membrane permeability, etc., are reported to be responsible for drug resistance in *C. albicans* [18]. To verify whether any such role of acquired mutations, a complementary strain *dpb3*ΔΔ::*DPB3* was generated by integrating a *DPB3* gene into its own locus of a *dpb3*ΔΔ strain of *C. albicans*. This was assessed for antifungal susceptibility, along with the wild-type and *dpb3*ΔΔ strains (Figure 4A). The spot analysis revealed that, while the *dpb3*ΔΔ was resistant to fluconazole, Amp B, 5FC, and caspofungin, the wild-type and complemented strains exhibited significant growth retardation. As Dpb3 is known to control the fidelity of Polε in *S. cerevisiae*, the *dpb3*ΔΔ strain of *C. albicans* may accumulate certain mutations in the genome; however, those mutations seem to have little or no effect on the multi-drug resistance phenotype. Thus, our result suggested that the function of Dpb3 or Polε *per se* is directly involved in the MDR phenotype of *C. albicans*.

### 3.4. Over-Expression of Hsp90 in Polε-Defective Strain of C. albicans

To decipher the possible molecular mechanism of azole resistance in Polε-defective strains of *C. albicans*, the expression of genes of drug efflux pumps, ergosterol biosynthesis, and heat-shock proteins was determined by using real-time and semi-quantitative RT-PCRs (Figure 4B,C). The increased expression of *CDR1*, *CDR2*, and *MDR1* genes is commonly found in azole-resistant clinical isolates of *Candida* species. Our expression analyses revealed that, except for *MDR1*, the mRNA level of the other efflux pumps, *CDR1* and *CDR2*, did not alter in the *dpb3*ΔΔ*dpb4*ΔΔ strain of *C. albicans*. However, *MDR1* mRNA expression decreased ~3-fold. Many azole-resistant *Candida* clinical isolates either show increased *ERG11* and *ERG3* expression or possess point mutations that cause non-functionality. Among the ergosterol biosynthesis genes, while the *ERG3* expression was reduced, the *ERG11* mRNA level remained the same in wild-type and *dpb3*ΔΔ*dpb4*ΔΔ strains of *C. albicans*. Heat-shock proteins (Hsps) are ubiquitous proteins expressed in response to various stresses and they play an important role in conferring resistance to antifungal drugs by regulating various signaling pathways in *C. albicans* [24]. Among the Hsps, it was striking to observe a high expression of *HSP90* mRNA (Figure 4B,C). To strengthen our result, the expression of Hsp90 protein was determined in the cell free lysates of wild-type and *dpb3*ΔΔ*dpb4*ΔΔ strains of *C. albicans* via probing with an anti-Hsp90 antibody (Figure 4D(i,ii)). Multiple repeats of the experiment confirmed an increased expression of Hsp90 in both mRNA and protein levels in the *dpb3*ΔΔ*dpb4*ΔΔ strain and a possible role in drug resistance. PCNA, a protein involved in DNA replication, was probed as a loading control [37].

### 3.5. Multi-Drug Resistance Phenotype of dpb3/dpb4 Null Strains of C. albicans Is Due to Cell Wall Deformity but Independent of Tor1 Signaling

**Figure 5:**
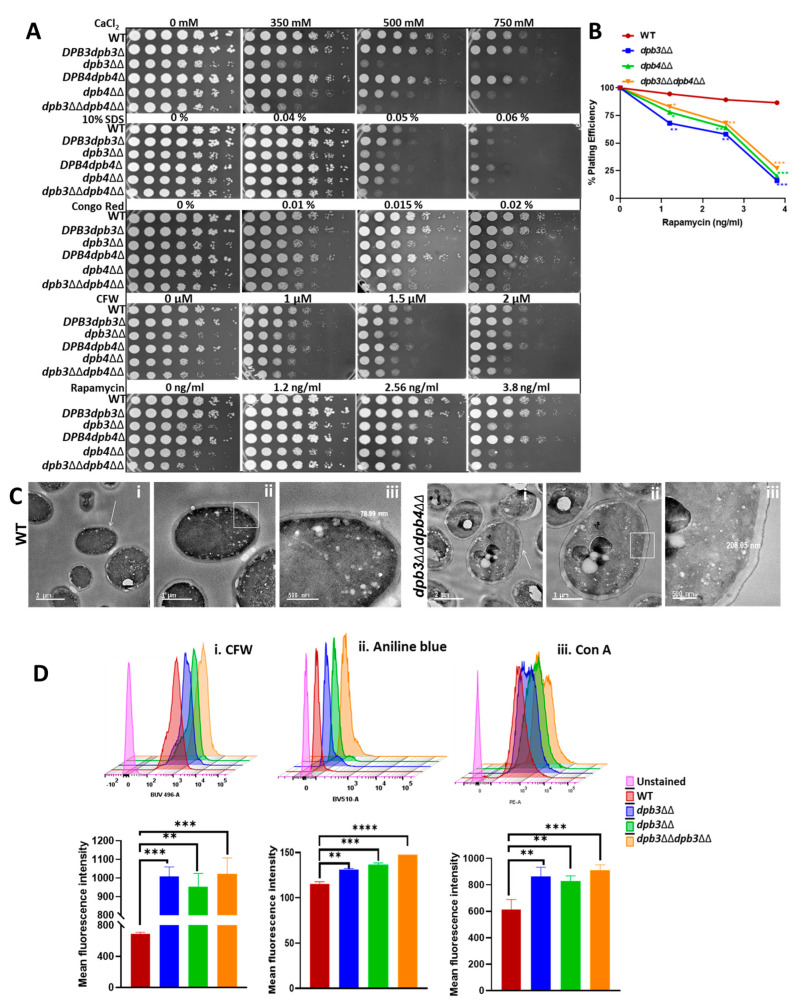
Xenobiotic drugs sensitivity and cell wall deformity. (**A**) Serially diluted cultures WT, *DPB3dpb3*Δ, *dpb3*ΔΔ, *DPB4dpb4*Δ, *dpb4*ΔΔ, and *dpb3*ΔΔ*dpb4*ΔΔ strains were spotted onto YPD plates without or with indicated concentrations of CaCl_2_, SDS, Congo red, CFW, and rapamycin. Plates were incubated at 30 °C for 48 h and photographed. (**B**) To confirm the effect of rapamycin, CFU analysis of these strains was carried out by spreading appropriate dilutions of cells onto a YPD plate containing different concentrations of rapamycin and counting the colony numbers after overnight incubation at 30 °C. The efficiency of CFU was analyzed using GraphPad Prism software version 8 and compared between WT and mutant strains using a two-way ANOVA test. (**C**) TEM images of ultra-thin sections of WT, and *dpb3*ΔΔ*dpb4*ΔΔ cells with scale bars = 2 μm (**i**), 1 μm (**ii**), and 500 nm (**iii**). The thickness of individual cell walls was measured. The arrow mark indicates the cell to be analyzed and the box indicates the zoom-in image of the area of the cell wall used to measure thickness. (**D**) Cells of various strains of *C. albicans* were stained with CFW (**i**), aniline blue (**ii**), and Con A (**iii**), and analyzed via flow cytometry. The mean fluorescence intensity from 3 independent experiments was plotted and compared between WT and mutant strains using a two-way ANOVA test. The results are presented as mean ± standard deviation and asterisks indicate the statistically significant differences (* *p* ≤ 0.1, ** *p* ≤ 0.01, *** *p* ≤ 0.001, and **** *p* ≤ 0.0001).

Changes in the cell wall of *C. albicans* can lead to poor drug absorption and are yet another mechanism of antifungal drug resistance. To check any possible changes in the cell wall structure and composition, the susceptibility of *C. albicans* cells was checked by exposing them to a range of cell wall-perturbing xenobiotics like sodium dodecyl sulfate (SDS), CaCl_2_, calcofluor white (CFW), and Congo red [38]. Surprisingly, unlike antifungal drugs, the growth of the homozygous deletion strains *dpb3*ΔΔ, *dpb4*ΔΔ, and *dpb3*ΔΔ*dpb4*ΔΔ was significantly reduced in the presence of the mentioned xenobiotics, while the growth of WT and heterozygous deletion strains was minimally perturbed (Figure 5A). Since there was no overexpression of drug efflux transporters and as genome instability contributed minimally to antifungal drug resistance, the MDR phenotype of *dpb3*/*dpb4*-deficient strains could likely be due to altered cell wall and Hsp90 function. To validate this again, we examined any modification in the cell wall structure and composition in *dpb3*ΔΔ*dpb4*ΔΔ *C. albicans* cells via transmission electron microscopy and flow cytometry analyses (Figure 5C,D). The TEM images revealed that the thickness of the cell wall (n = 3) of *dpb3*ΔΔ*dpb4*ΔΔ *C. albicans* cells (177.5 ± 22 nm) was about 2-fold more than that of WT cells (86.2 ± 5.5 nm) (Figure 5C and Supplementary Table S2). The cell wall of *C. albicans* had an inner chitin layer, a middle β-glucans layer, and mannan as the outermost layer [39]. While chitin level was estimated via CFW staining of the cells, β-glucan and mannan levels were measured by staining with aniline blue and concanavalin A tetramethylrhodamine dyes, respectively, and the mean fluorescence intensity of various stained cells was measured via flow cytometry (Figure 5D(i–iii) and Supplementary Table S3). While the chitin content was ~2-fold more in the strains without *DPB3*, *DPB4*, or both the subunits, other layers were marginally increased compared to that in WT. These results suggested a clear alteration in the cell wall structure and composition of Polε-defective strains of *C. albicans* cells, which could be one of the reasons for the low adsorption of antifungal drugs. At the same time, a higher chitin content will facilitate more binding of CFW and Congo red, causing Polε-defective strains to be more susceptible than WT. Since we observed altered cell wall and overexpression of Hsp90 in *dpb3*ΔΔ*dpb4*ΔΔ strain, and as both were linked by the target of rapamycin (Tor1) signaling, we wanted to explore the possible role of Tor1 hyperactivation in azole-resistant cells. As rapamycin inhibits Tor1 hyperactivation, we compared the growth of various strains in the presence of rapamycin and found that the *dpb3*/*dpb4*-deficient strains were more sensitive than the WT and heterozygous deletion strains (Figure 5A). To reconfirm, CFU analysis was carried out in the presence of different concentrations of rapamycin and a similar result was obtained, where the *dpb3*ΔΔ, *dpb4*ΔΔ, and *dpb3*ΔΔ*dpb4*ΔΔ strains developed poor colony numbers in the presence of rapamycin (Figure 5B). The sensitivity of Polε-defective strains to rapamycin suggested the absence of Tor1 hyperactivation, and Hsp90 could play a role in azole drug resistance independently of Tor1 signaling.

### 3.6. Hsp90 Function Is Critical for Multi-drug Resistance in C. albicans

**Figure 6:**
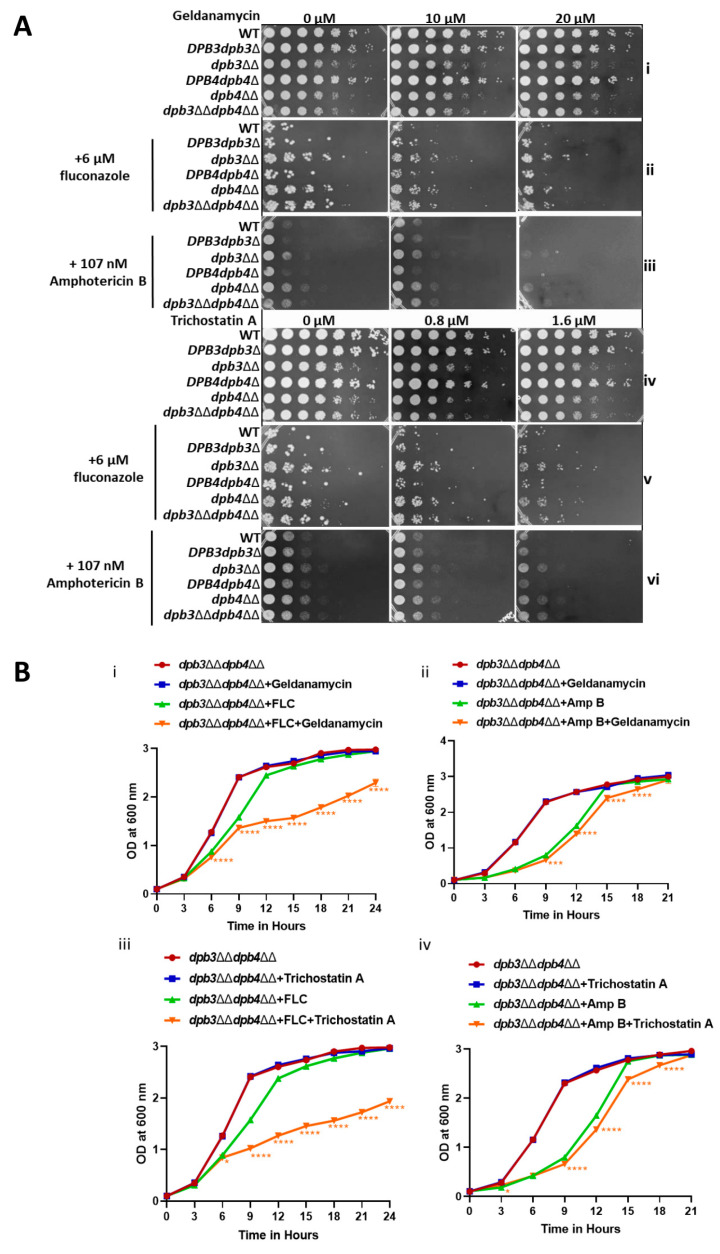
Hsp90 inhibitors sensitize antifungal drug sensitivity. (**A**) Serially diluted cultures of WT, *DPB3dpb3*Δ, *dpb3*ΔΔ, *DPB4dpb4*Δ, *dpb4*ΔΔ, and *dpb3*ΔΔ*dpb4*ΔΔ strains were spotted onto YPD plates containing either indicated concentration of geldanamycin, trichostatin A, FLC, Amp B, and combinations thereof. All the plates were incubated at 30 °C for 48 h and photographed. (**B**) The absorbance of these strains while growing in YPD media in the presence or absence of FLC alone, AmpB alone, geldanamycin alone, trichostatin A alone, and in combinations at 30 °C, was recorded and plotted using GraphPad Prism 8.0 and compared between single- (green) and double-drug (orange) treatments using a two-way ANOVA test. The results are presented as mean ± standard deviation and asterisks indicate the statistically significant differences between strains treated with single- and double-drug combinations in each graph. The statistically significant differences (* *p* ≤ 0.05, *** *p* ≤ 0.001, and **** *p* < 0.0001) between the results of WT and mutant strains were determined by using a two-way ANOVA test (Dunnett’s multiple comparisons).

Hsp90 plays an important role in antifungal drug resistance by regulating signaling cascades like calcium–calcineurin, MAPK, Ras1-cAMP-PKA, and cell cycle control signaling in *C. albicans* [6,24], and recent reports suggest that azole resistance can be reversed by inhibiting the Hsp90 function [40,41,42,43]. We argued that if the azole resistance by *dpb3*ΔΔ*dpb4*ΔΔ strain was indeed due to the hyperactivation of Hsp90, inhibitors of Hsp90’s function could sensitize the resistant *C. albicans* cells to azoles. Geldanamycin is a competitive inhibitor that binds to the ADP/ATP-binding site of Hsp90. The acetylation of Hsp90 is critical for its role in the emergence of azole resistance and trichostatin A prevents the deacetylation of Hsp90 by inhibiting the respective lysine deacetylase enzyme [44]. We performed spot and growth curve assays in the presence of Hsp90 inhibitors, FLC or Amp B alone, or a combination of these reagents (Figure 6A,B and Supplementary Table S4). In both assays, while geldanamycin and trichostatin A alone did not affect the survival of *C. albicans* strains (panels Figure 6A(i,iv)), the presence of these inhibitors enhanced the sensitivity of resistant cells to a mild dosage of fluconazole and amphotericin B (panels Figure 6A(ii,iii,v,vi)). The growth curves of drug resistance strains *dpb3*ΔΔ, *dpb4*ΔΔ, and *dpb3*ΔΔ*dpb4*ΔΔ were significantly reduced in the combinatorial treatment in comparison to azole/Amp B drug alone (Figure 6B). Altogether, our results confirm that the Hsp90 function is critical to the antifungal drug resistance of *dpb3*/*dpb4*-deficient strains.

### 3.7. Loss of Dpb3 and Dpb4 Subunits Induces Biofilm Formation of C. albicans

**Figure 7:**
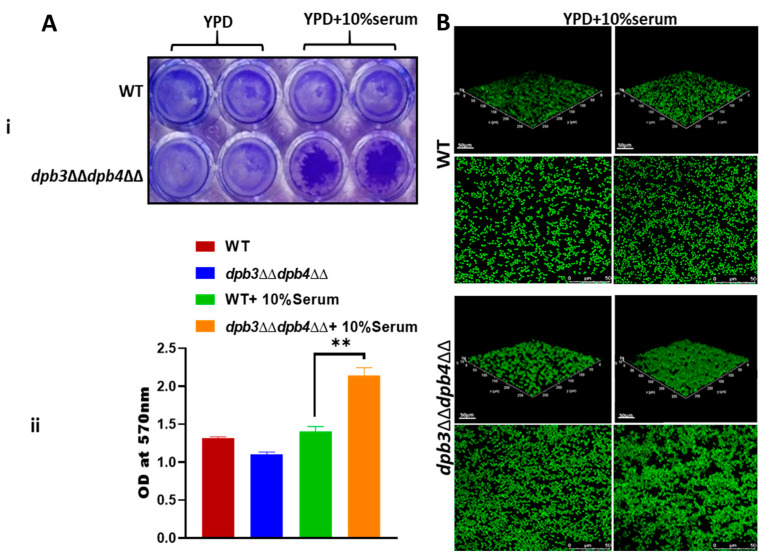
Biofilm detection. (**A**) The WT and *dpb3*ΔΔ*dpb4*ΔΔ strains of *C. albicans* (n = 2) were grown in YPD media without and with 10% FBS in 24-well polystyrene plates for 24 h at 30 °C. The wells were washed with PBS and stained with 0.05% crystal violet to visualize biofilm (**i**) and their absorbance was measured at 570 nm (**ii**). The obtained absorbance was plotted. The results are presented as mean ± standard deviation and asterisks indicate the statistically significant differences between the samples (** *p* ≤ 0.01), as determined using a one-way ordinary ANOVA test (Dunnett’s multiple comparisons). (**B**) Similarly grown *C. albicans* cells (n = 2) on a 6-wells polystyrene plate containing glass coverslips for 24 h at 30 °C. The biofilm formed on the coverslip was stained with 1% acridine orange and images were captured using Leica TCS SP8 confocal system with an excitation wavelength of 483 nm and emission wavelength of 510 nm. The upper panel is a 3D image, and the lower panel is a 2D image.

Biofilm-forming strains are recalcitrant to antifungal susceptibility. It has been suggested that the biofilm grown on medical devices is the leading cause of chronic fungal infections in humans [4]. The fungal cells released from a matured biofilm appear to be highly virulent compared to planktonic cells in terms of systemic candidiasis development [45,46] and Hsp90 also plays an important role in biofilm dispersions [44,47]. Since we could find the overexpression of Hsp90 and its function in azole drug resistance, we verified the biofilm formation ability of *dpb3*/*dpb4*-deficient strains on polystyrene surfaces by crystal violet staining and confocal scanning laser microscopy (CSLM) (Figure 7). In the absence of serum, both WT and *dpb3*ΔΔ*dpb4*ΔΔ strains developed a similar level of biofilm; however, serum induced robust biofilm via the *dpb3*ΔΔ*dpb4*ΔΔ rather than the WT strain (Figure 7A(i,ii)). We further confirmed the morphology of the biofilm via CSLM, using silicone squares as the base, and found that while the wild-type strain formed a thin layer of biofilm, the *dpb3*ΔΔ*dpb4*ΔΔ strain developed thicker, compact, and aggregated biofilm (Figure 7B).

## 4. Discussion

**Figure 8:**
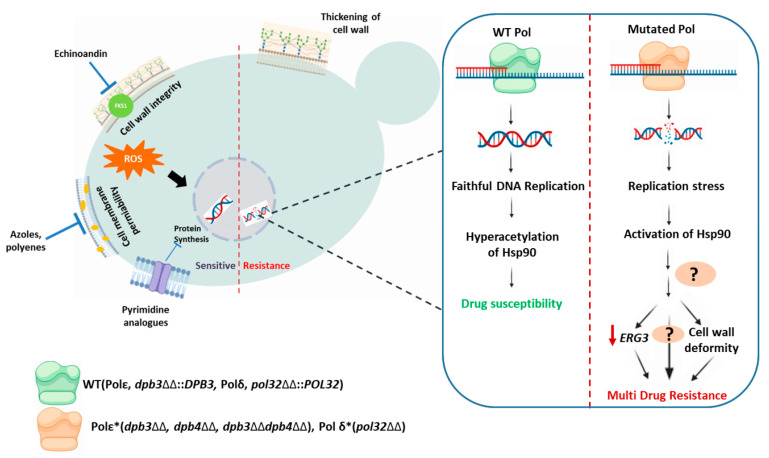
A uniform model of drug resistance mechanism in replication-defective strains of *C. albicans.* Replication stress due to a defect in the DNA polymerase subunit’s function induces Hsp90 expression and activity, causing antifungal drug resistance that is independent of Tor1 signaling. Cell wall deformity with high chitin content and *ERG3* downregulation can be a consequence of the Hsp90 stress response that inhibits drug adsorption and toxic sterol production, respectively, to induce drug resistance. * indicates defective in function and arrow denotes down-regulation (curtsey of BioRender).

The occurrence of fungal infections is currently on the rise due to an increase in the number of immune-compromised individuals and due to an increase in the ever-evolving drug-resistant fungal isolates, which is contributing to a high load of morbidity and mortality worldwide [1]. The identification of azole-resistant clinical isolates is very frequent in comparison to other antifungal drugs [48]. In this study, we have identified and characterized three different knockout strains of *C. albicans* that are defective in accessory subunits of Polε, a DNA polymerase involved in several processes of genome stability, including initiating and leading strand DNA synthesis during DNA replication [25,49]. We showed that the loss of small subunits of Polε (Dpb3 and Dpb4) caused the strains to develop resistance, not only to the azole group, but also to non-azole groups of clinically relevant drugs like Amp B, 5FC, and caspofungin, and to develop robust biofilms. These strains showed weak but significant resistance to 5+FC and caspofungin. Interestingly, following the deletion of any of the subunits of Polε, whether it was Dpb3 or Dpb4, the strains exhibited similar growth defects and drug resistance phenotypes, suggesting that both subunits are important and function via the same pathway. Further, we explored possible mechanisms of drug resistance in these laboratory isolates. Several molecular mechanisms have been reported to allow *C. albicans* to develop antifungal drug resistance [6,18,50]. Several examples of such mechanisms include structural alterations of the drug target, the overexpression of target gene products, efficient drug expulsion due to the overexpression of drug efflux transporters, and changes in the composition and architecture of cell walls and cell membranes. Insertions, deletions, and single-nucleotide polymorphisms in gene encoding targets and their regulatory proteins, and higher-order chromosomal instability issues such as aneuploidy and isochromosome formation are commonly associated with drug resistance in *Candida*. The hyperactivation of the target of rapamycin (TOR) signaling is another mechanism in *C. albicans* for bypassing azole toxicity. Most antifungals generate oxidative stress in the cells; therefore, the overexpression of certain genes that reduce oxidative damage acts as a counteractive measure on strains to suppress antifungal susceptibility [51]. Considering that the evolutionarily conserved role of Polε in genome stability and the deletion of *DPB3* and *DPB4* genes is known to enhance mutagenesis in budding yeast, one would expect the accumulation of mutations in the genome due to replication errors that may induce drug resistance. However, the integration of a copy of *DPB3* into the genome of the *dpb3*ΔΔ strain reversed the phenotype from one of drug resistance to sensitivity. Again, we did not observe the overexpression of any of the important drug transporters (*CDR1*, *CDR2*, and *MDR1*). Rapamycin resistance suggests the hyperactivation of *TOR1* in azole-resistant isolates [52]; however, we here observed the Polε-defective strains to be hypersensitive to rapamycin. Altogether, our results ruled out the critical involvement of any of the above-discussed mechanisms in the drug resistance of Polε-defective strains. More importantly, we observed altered cell wall structure and composition. A reduced level of cellular ROS species accumulation means less oxidative stress, lower expression of *ERG3*, and increased expression of Hsp90 in *dpb3*ΔΔ/*dpb4*ΔΔ strains (Figure 8). Hsps are ubiquitous proteins usually expressed in response to thermal stress; however, studies also show their activation to non-thermal stressors such as heavy metals, oxidative stress, and genomic instability [53]. An interactome analysis suggested the role of Hsp90 in DNA metabolism and cell division in budding yeast [54]. A more recent report suggested a direct link between Hsp90 and cell cycle progression as it stabilizes the E2F1 transcription factor in order to regulate the expression of cell cycle-related proteins such as cell division cycle 6 (CDC6), cell division cycle 45 (CDC45), minichromosome maintenance 4 (MCM4), minichromosome maintenance 7 (MCM7), RecQ-mediated genome instability 2, and DNA primase polypeptide 1 [55]. Several studies suggested a direct correlation between Hsp90 function and genome stability; thus, the hyperexpression of this protein in Polε-defective strain is not surprising, although a molecular mechanism is yet to be established [56,57]. Hsp90 hyperexpression and the inhibition of its function, both directly by geldanamycin and indirectly by trichostatin A sensitizing to azole and Amp B, unequivocally supported the critical role of Hsp90 in the drug resistance of Polε-defective strains. Lower drug adsorption due to altered cell wall composition and Hsp90 function seem to be the main reasons for drug resistance, and they may also be interconnected. The disruption of *ERG3* has also been reported, leading to azole drug resistance but hypersensitivity to Hsp90 inhibitor geldanamycin [58,59]. Since Hsp90 functions downstream of Tor1 signaling and as Tor1 seems to be less active in our case, the exact mechanism of Hsp90 action, causing drug resistance in Polε-defective strains independently of Tor1, requires further investigation. Hsp90 most likely organizes a cellular stress response circuit upon registering replication stress, which has a major impact on resistance to several antifungals. The stress response by Hsp90 is regulated by several factors like the affinity of ATP binding and hydrolysis, interactions with co-chaperones, and post-translational modifications. The role of lysine deacetylases (KDACs) like Hda1 and Rpd3 in regulating Hsp90 function has been reported in azole resistance in *C. albicans* and *S. cerevisiae*. The inhibition of lysine deacetylases by molecules like trichostatin A leads to the accumulation of hyperacetylated Hsp90. Hyperacetylation prevents the binding of co-chaperons like calcineurin and other client proteins, thereby inhibiting Hsp90’s function [60].

In a parallel study, we recently reported that the *pol32*-defective strain showed azole drug resistance but amphotericin B sensitivity [27]. Although the whole-genome sequencing of the *pol32*ΔΔ strain revealed an accumulation of indels and SNPs, the acquired mutations had either no or little effect on azole resistance as the *pol32*ΔΔ::*POL32* strain was sensitive to azoles. Additionally, altered cell wall architecture, with a higher amount of chitin and glucan, and the reduced expression of *ERG3* were also observed to be associated with azole drug resistance in *pol32*ΔΔ. Similar to Dpb3/Dpb4-null strains, the mechanism of azole drug resistance of *pol32*ΔΔ was mostly due to Hsp90 function but was independent of Tor1 signaling as the *pol32*ΔΔ cells were also sensitive to rapamycin. Geldanamycin and trichostatin A treatment sensitized the *pol32*ΔΔ strain to azole. Replication and oxidative stresses are known to upregulate *HSP90* expression. Although antifungal drugs also enhance oxidative stress, this overexpression of *HSP90* is most likely due to the replication stress in both Polδ- and Polε-defective strains [51,61]. Considering all our observations, we propose a uniform mode of antifungal drug resistance in *C. albicans* and suggest that replication stress due to defect in DNA polymerase function induces Hsp90 expression and that the activity of Hsp90 causes antifungal drug resistance that is independent of Tor1 signaling (Figure 8). Cell wall deformity with high chitin content and *ERG3* downregulation could be a consequence of an Hsp90 stress response that inhibits drug adsorption and toxic sterol production, respectively, in order to induce drug resistance. Pharmacological failures in combating fungal infections have drawn attention to addressing the problems of antifungal resistance, deciphering underlying mechanisms, and exploring new areas of drug targets. This study and earlier reports found the increased reversal of azole drug resistance to be due to geldanamycin and trichostatin A treatment [27,41,42]. This reconfirms that the use of a combinatorial therapy by targeting Hsp90 and its upstream and downstream targets enhance the efficacy of readily available drugs.

## Figures and Tables

**Table 1:** Primers used for real-time PCR.

Gene Name	Forward Primer	Reverse Primer
*GAPDH*	5′-gaccgttgacggtccatcc-3′	5′-catcggtggttgggactc-3′
*CDR1*	5′-aaagatgacctcgtcagcaggttt-3′	5′-ccaattcccaatttcgaaggt-3′
*CDR2*	5′-tgttggtaccatttcatatttctgttg-3′	5′-aagagattgccaattgtcccata-3′
*MDR1*	5′-tcgttttagcaatggcgtttg-3′	5′-ccatgccctccaatgaacag-3′
*ERG3*	5′-tccagttgatgggttcttcc-3′	5′-ggacagtgtgacaagcgg-3′
*ERG11*	5′-ttacctcattattggagacgtgatg-3′	5′-cacgttctcttctcagtttaatttctttc-3′
*HSP90*	5′-aagtgctggtgctgacg-3′	5′-cttaccaccagcgttag-3′
*HSP30*	5′-catgctccaactgctac-3′	5′-cgttcttcagcttcggc-3′

## Data Availability

Data are contained within the article and Supplementary Materials.

## Supplementary Materials

The following supporting information can be downloaded at: https://www.mdpi.com/article/10.3390/jof10030222/s1.
