# Supplementary material for: Hsp90-Mediated Multi-Drug Resistance in DNA Polymerase-Defective Strains of Candida albicans
Source: J Fungi (Basel). 2024 Mar 19;10(3):222. doi: 10.3390/jof10030222 (PMC10970858; doi:10.3390/jof10030222)
Supplement: Supplementary file 1 [file jof-10-00222-s001.zip › jof-2885039-supplementary.pdf]

<u>Supplementary Information</u>

Article

Hsp90-Mediated Multi-Drug Resistance in DNA Polymerase-Defective Strains of *Candida albicans*

Bhabasha Gyanadeep Utkalaja [1,2], Satya Ranjan Sahu [1,2], Sushree Subhashree Parida [1,2] and Narottam Acharya [1,*]

[1] Laboratory of Genomic Instability and Diseases, Department of Infectious Disease Biology, Institute of Life Sciences, Bhubaneswar 751023, India

[2] Regional Center of Biotechnology, Faridabad 121001, India

* Correspondence: narottam_acharya@ils.res.in or narottam74@gmail.com; Tel.: +91-674-230-4278; Fax: +91-674-230-0728

Running title: Role of Hsp90 in drug resistance

Keywords: DNA replication, DNA polymerase epsilon, *Candida*, Candidiasis, Hsp90, azoles, drug resistance, biofilm

Supplementary Figure S1: (**A**). Overnight cultures of WT, *DPB3dpb3*Δ, *dpb3*ΔΔ, *DPB4dpb4*Δ, *dpb4*ΔΔ, and *dpb3*ΔΔ*dpb4*ΔΔ strains were serially diluted and spotted on YPD plates without or with the indicated concentrations of 5FC and 5FU. All the plates were incubated at 30 °C for 48 hr and photographed. (**B**) Growth curve analyses of these strains were carried out in the presence of 5-FC (50 μM, i). The absorbance was measured at OD_{600} for 21 hr at a regular interval (Supp. Table -5) and was plotted using GraphPad Prism 8.0. **(C)** Drug accumulation assay was carried by adding berberine to 5 x 10^7 *C. albicans* cells in 1 x PBS and incubated at 30 °C in 200 rpm shaking condition. Cells were collected at regular interval and fluorescence was measured in an ELISA plate reader with the excitation and emission wavelength of 360 nm and 520 nm, respectively. Asterisks indicate (****$P \leq 0.0001$, ***$P \leq 0.001$, **$P \leq 0.01$, and *$P<0.05$) the statistically significant differences between the results of WT and mutant strains using a two-way ANOVA test.

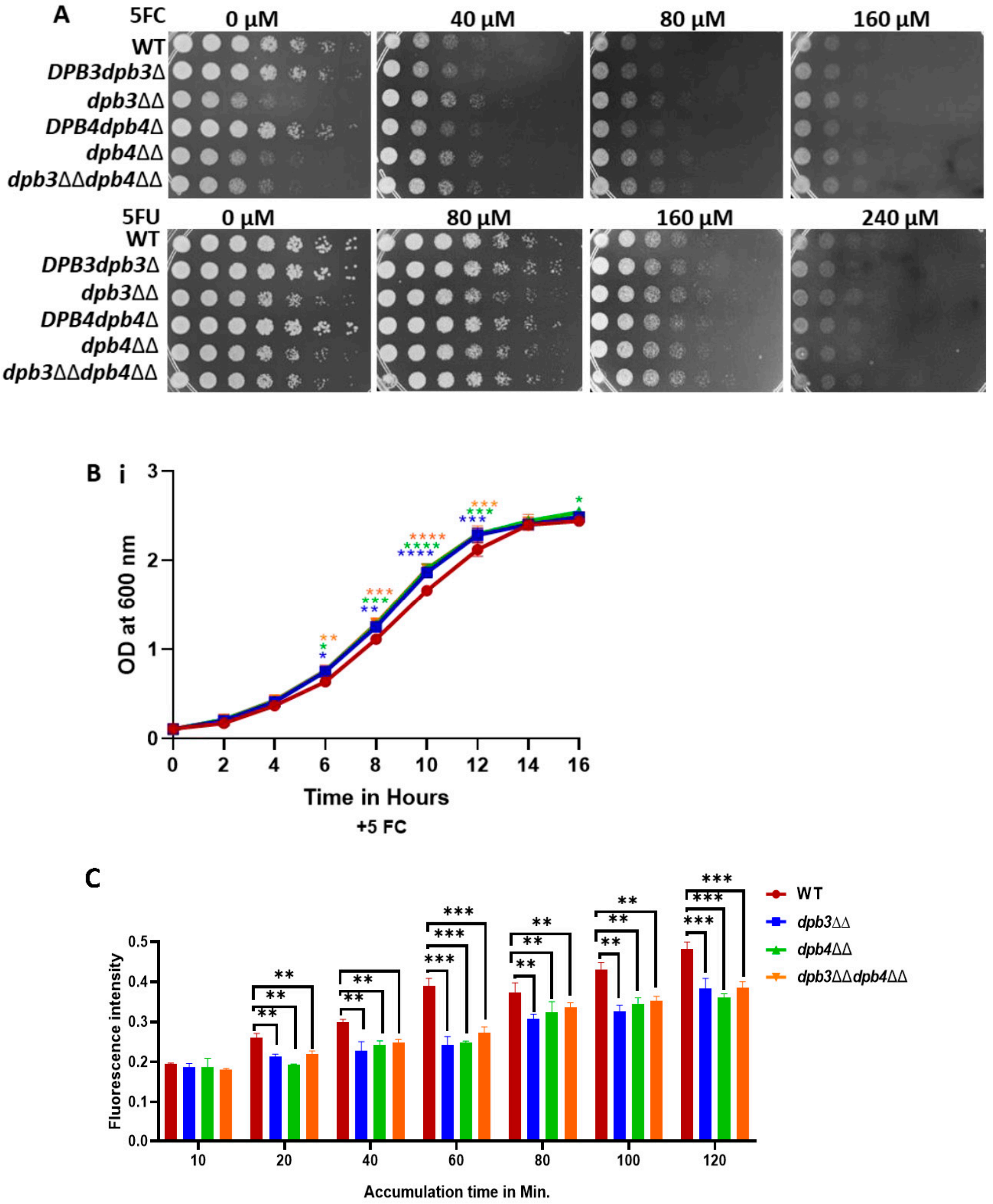
A
5FC
0 μM
40 μM
80 μM
160 μM
WT
DPB3dpb3Δ
dpb3ΔΔ
DPB4dpb4Δ
dpb4ΔΔ
dpb3ΔΔdpb4ΔΔ
5FU
0 μM
80 μM
160 μM
240 μM
B i
OD at 600 nm
Time in Hours
+5 FC
C
Fluorescence intensity
Accumulation time in Min.
WT
dpb3ΔΔ
dpb4ΔΔ
dpb3ΔΔdpb4ΔΔ

Supplementary Table-S1: The growth curve analyses of WT, *dpb3*ΔΔ, *dpb4*ΔΔ, and *dpb3*ΔΔ*dpb4*ΔΔ strains were carried out without (**a**) and with fluconazole (**b**), amphotericin B (**c**), and caspofungin (**d**). The absorbance measured at different time points and statistical analysis has been given as tables.

(**a**) without any drugs

														**P value**		
	**Time(hrs)**	**WT**	**Average**	**Average of averages ± SD**	***dpb3*ΔΔ**	**Average**	**Average of averages ± SD**	***dpb4*ΔΔ**	**Average**	**Average of averages ± SD**	***dpb3*ΔΔ *dpb4*ΔΔ**	**Average**	**Average of averages ± SD**	***WT vs dpb3ΔΔ***	***WT vs dpb4ΔΔ***	***WT vs dpb3ΔΔ dpb4ΔΔ***
		0.101			0.102			0.103			0.104					
**Set 1**	0	0.098	0.0995		0.106	0.104		0.107	0.105		0.099	0.1015				
		0.106		0.103±0.0035	0.103		0.104±0.001	0.105		0.10575±0.0007	0.105		0.10275±0.0012			
**Set 2**	0	0.107	0.1065		0.105	0.104		0.108	0.1065		0.103	0.104		>0.9999	0.9983	>0.9999
		0.287			0.249			0.243			0.253					
**Set 1**	2	0.291	0.289		0.274	0.2615		0.265	0.254		0.268	0.2605				
		0.297		0.2935±0.0045	0.256		0.262±0.005	0.256		0.2495±0.0045	0.239		0.25775±0.0027			
**Set 2**	2	0.299	0.298		0.269	0.2625		0.234	0.245		0.271	0.255		0.3147	0.1058	0.2238
		0.801			0.613			0.574			0.588					
**Set 1**	4	0.789	0.795		0.587	0.6		0.581	0.5775		0.549	0.5685				
		0.79		0.79475±0.00025	0.601		0.591±0.009	0.587		0.57275±0.004	0.578		0.57725±0.0087			
**Set 2**	4	0.799	0.7945		0.563	0.582		0.549	0.568		0.594	0.586		<0.0001	<0.0001	<0.0001
		1.765			1.313			1.265			1.265					
**Set 1**	6	1.819	1.792		1.298	1.3055		1.297	1.281		1.285	1.275				
		1.762		1.79025±0.0017	1.323		1.308±0.0025	1.198		1.27275±0.008	1.242		1.25675±0.0182			
**Set 2**	6	1.815	1.7885		1.298	1.3105		1.331	1.2645		1.235	1.2385		<0.0001	<0.0001	<0.0001
		2.734			2.207			2.259			2.291					
**Set 1**	8	2.845	2.7895		2.224	2.2155		2.249	2.254		2.285	2.288				
		2.66		2.756±0.0335	2.195		2.20725±0.0082	2.276		2.22325±0.030	2.327		2.27875±0.0092			
**Set 2**	8	2.785	2.7225		2.203	2.199		2.109	2.1925		2.212	2.2695		<0.0001	<0.0001	<0.0001
		3.119			2.632			2.532			2.548					
**Set 1**	10	3.167	3.143		2.597	2.6145		2.56	2.546		2.532	2.54				
		3.124		3.13575±0.0072	2.532		2.5875±0.027	2.514		2.52125±0.024	2.538		2.513±0.027			
**Set 2**	10	3.133	3.1285		2.589	2.5605		2.479	2.4965		2.434	2.486		<0.0001	<0.0001	<0.0001
		3.298			3.218			3.278			3.217					
**Set 1**	12	3.347	3.3225		3.279	3.2485		3.254	3.266		3.298	3.2575				
		3.314		3.32±0.0025	3.198		3.24575±0.027	3.224		3.24025±0.025	3.204		3.247±0.0105			
**Set 2**	12	3.321	3.3175		3.288	3.243		3.205	3.2145		3.269	3.2365		0.0033	0.0016	0.0038
		3.376			3.31			3.367			3.331					
**Set 1**	14	3.357	3.3665		3.317	3.3135		3.373	3.37		3.365	3.348				
		3.354		3.36275±0.0037	3.276		3.301±0.0125	3.34		3.35775±0.012	3.28		3.31925±0.0287			
**Set 2**	14	3.364	3.359		3.301	3.2885		3.351	3.3455		3.301	3.2905		0.0154	0.9903	0.111

(b) with fluconazole

														P value		
	Time(hrs)	WT	Average	Average of averages ± SD	*dpb3*ΔΔ	Average	Average of averages ± SD	*dpb4*ΔΔ	Average	Average of averages ± SD	*dpb3*ΔΔ *dpb4*ΔΔ	Average	Average of averages ± SD	***WT vs dpb3ΔΔ***	***WT vs dpb4ΔΔ***	***WT vs dpb3ΔΔ dpb4ΔΔ***
**Set 1**	0	0.103<br>0.105	0.104		0.109<br>0.104	0.1065		0.108<br>0.103	0.1055		0.11<br>0.108	0.109				
**Set 2**	0	0.1<br>0.098	0.099	0.1015±0.002	0.105<br>0.101	0.103	0.10475±0.0017	0.105<br>0.102	0.1035	0.1045±0.001	0.108<br>0.106	0.107	0.108±0.001	0.995	0.9961	0.9635
**Set 1**	6	0.843<br>0.901	0.872		0.853<br>0.886	0.8695		0.873<br>0.923	0.898		0.869<br>0.914	0.8915				
**Set 2**	6	0.891<br>0.886	0.8885	0.1015±0.008	0.876<br>0.891	0.8835	0.8765±0.007	0.854<br>0.937	0.8955	0.89675±0.001	0.871<br>0.893	0.882	0.88675±0.004	0.9925	0.65	0.9635
**Set 1**	9	0.984<br>1.031	1.0075		1.246<br>1.273	1.2595		1.259<br>1.293	1.276		1.263<br>1.301	1.282				
**Set 2**	9	0.976<br>0.996	0.986	0.1015±0.010	1.239<br>1.261	1.25	1.25475±0.004	1.276<br>1.287	1.2815	1.27875±0.002	1.259<br>1.298	1.2785	1.28025±0.001	<0.0001	<0.0001	<0.0001
**Set 1**	12	1.143<br>1.053	1.098		1.286<br>1.353	1.3195		1.339<br>1.326	1.3325		1.373<br>1.254	1.3135				
**Set 2**	12	1.159<br>1.117	1.138	0.1015±0.02	1.312<br>1.345	1.3285	1.324±0.004	1.359<br>1.298	1.3285	1.3305±0.002	1.369<br>1.324	1.3465	1.33±0.016	<0.0001	<0.0001	<0.0001
**Set 1**	15	1.273<br>1.148	1.2105		1.473<br>1.398	1.4355		1.456<br>1.432	1.444		1.473<br>1.429	1.451				
**Set 2**	15	1.265<br>1.225	1.245	0.1015±0.0172	1.457<br>1.415	1.436	1.43575±0.0002	1.476<br>1.421	1.4485	1.44625±0.002	1.487<br>1.446	1.4665	1.45875±0.007	<0.0001	<0.0001	<0.0001
**Set 1**	18	1.253<br>1.283	1.268		1.523<br>1.489	1.506		1.514<br>1.462	1.488		1.541<br>1.483	1.512				
**Set 2**	18	1.298<br>1.304	1.301	0.1015±0.0165	1.559<br>1.529	1.544	1.525±0.019	1.545<br>1.518	1.5315	1.50975±0.021	1.562<br>1.503	1.5325	1.52225±0.0102	<0.0001	<0.0001	<0.0001
**Set 1**	21	1.334<br>1.303	1.3185		1.643<br>1.632	1.6375		1.673<br>1.612	1.6425		1.694<br>1.623	1.6585				
**Set 2**	21	1.319<br>1.354	1.3365	0.1015±0.009	1.679<br>1.703	1.691	1.66425±0.026	1.698<br>1.639	1.6685	1.6555±0.013	1.65<br>1.641	1.6455	1.652±0.0065	<0.0001	<0.0001	<0.0001
**Set 1**	24	1.394<br>1.383	1.3885		1.743<br>1.714	1.7285		1.798<br>1.698	1.748		1.763<br>1.713	1.738				
**Set 2**	24	1.379<br>1.391	1.385	0.1015±0.0017	1.734<br>1.754	1.744	1.73625±0.0077	1.81<br>1.768	1.789	1.7685±0.0205	1.798<br>1.773	1.7855	1.76175±0.023	<0.0001	<0.0001	<0.0001

(**c**) with amphotericin b

														P value		
	Time(hrs)	WT	Average	Average of averages ± SD	*dpb3*ΔΔ	Average	Average of averages ± SD	*dpb4*ΔΔ	Average	Average of averages ± SD	*dpb3*ΔΔ *dpb4*ΔΔ	Average	Average of averages ± SD	*WT vs dpb3ΔΔ*	*WT vs dpb4ΔΔ*	*WT vs dpb3ΔΔ dpb4ΔΔ*
		0.097			0.117			0.096			0.112					
**Set 1**	0	0.1	0.0985		0.105	0.111		0.104	0.1		0.108	0.11				
		0.104			0.105			0.103			0.105					
**Set 2**	0	0.103	0.1035	0.101 ± 0.002	0.102	0.1035	0.10725± 0.003	0.11	0.1065	0.10325± 0.003	0.107	0.106	0.108± 0.002	0.9949	0.9997	0.9929
		0.125			0.187			0.127			0.15					
**Set 1**	3	0.121	0.123		0.165	0.176		0.133	0.13		0.157	0.1535				
		0.135			0.191			0.145			0.164					
**Set 2**	3	0.145	0.14	0.1315 ± 0.009	0.172	0.1815	0.17875± 0.002	0.165	0.155	0.1425±0.012	0.171	0.1675	0.1605± 0.007	0.3411	0.9738	0.7008
		0.15			0.268			0.181			0.182					
**Set 1**	6	0.158	0.154		0.26	0.264		0.185	0.183		0.176	0.179				
		0.176			0.271			0.211			0.245					
**Set 2**	6	0.185	0.1805	0.16725 ± 0.013	0.289	0.28	0.272± 0.008	0.234	0.2225	0.20275± 0.019	0.267	0.256	0.2175± 0.038	0.0073	0.5635	0.2942
		0.218			0.406			0.258			0.272					
**Set 1**	9	0.211	0.2145		0.389	0.3975		0.261	0.2595		0.263	0.2675				
		0.23			0.398			0.382			0.298					
**Set 2**	9	0.254	0.242	0.22825 ± 0.013	0.437	0.4175	0.4075± 0.01	0.345	0.3635	0.3115±0.052	0.363	0.3305	0.299± 0.031	<0.0001	0.0381	0.0897
		0.325			0.634			0.647			0.81					
**Set 1**	12	0.365	0.345		0.589	0.6115		0.741	0.694		0.912	0.861				
		0.372			0.687			0.679			0.789					
**Set 2**	12	0.394	0.383	0.364 ± 0.019	0.659	0.673	0.64225± 0.030	0.712	0.6955	0.69475± 0.0007	0.783	0.786	0.8235± 0.037	<0.0001	<0.0001	<0.0001
		0.772			1.152			1.156			1.214					
**Set 1**	15	0.662	0.717		1.231	1.1915		1.197	1.1765		1.452	1.333				
		0.757			1.257			1.324			1.317					
**Set 2**	15	0.716	0.7365	0.72675 ± 0.0009	1.298	1.2775	1.2345± 0.043	1.298	1.311	1.24375± 0.067	1.386	1.3515	1.34225± 0.009	<0.0001	<0.0001	<0.0001
		1.432			2.532			2.432			2.887					
**Set 1**	18	1.398	1.415		2.641	2.5865		2.394	2.413		2.485	2.686				
		1.454			2.567			2.367			2.798					
**Set 2**	18	1.378	1.416	1.4155 ± 0.0005	2.521	2.544	2.56525± 0.021	2.484	2.4255	2.41925± 0.006	2.518	2.658	2.672± 0.014	<0.0001	<0.0001	<0.0001
		2.841			2.846			2.848			2.881					
**Set 1**	21	2.732	2.7865		2.883	2.8645		2.832	2.84		2.848	2.8645				
		2.654			2.798			2.859			2.889					
**Set 2**	21	2.839	2.7465	2.7665 ± 0.02	2.865	2.8315	2.848± 0.016	2.877	2.868	2.854±0.014	2.845	2.867	2.86575± 0.001	0.0432	0.0279	0.0113
		2.885			2.863			2.863			2.893					
**Set 1**	24	2.739	2.812		2.841	2.852		2.889	2.876		2.849	2.871				
		2.898			2.863			2.898			2.895					
**Set 2**	24	2.879	2.8885	2.85025 ± 0.038	2.876	2.8695	2.86075± 0.008	2.886	2.892	2.884±0.008	2.871	2.883	2.877± 0.006	0.977	0.6002	0.7468

(**d**) with caspofungin

														P value		
	Time(hrs)	WT	Average	Average of averages ± SD	*dpb3*ΔΔ	Average	Average of averages ± SD	*dpb4*ΔΔ	Average	Average of averages ± SD	*dpb3*ΔΔ *dpb4*ΔΔ	Average	Average of averages ± SD	*WT vs dpb3ΔΔ*	*WT vs dpb4ΔΔ*	*WT vs dpb3ΔΔ dpb4ΔΔ*
		0.103			0.109			0.108			0.11					
**Set 1**	0	0.105	0.104		0.104	0.1065		0.103	0.1055		0.108	0.109				
		0.1			0.095			0.102			0.106					
**Set 2**	0	0.108	0.104	0.104	0.106	0.1005	0.1035±0.003	0.104	0.103	0.10425± 0.0012	0.102	0.104	0.1065±0.002	>0.9999	>0.9999	0.9997
		0.401			0.489			0.494			0.473					
**Set 1**	8	0.451	0.426		0.501	0.495		0.521	0.5075		0.513	0.493				
		0.389			0.459			0.51			0.495					
**Set 2**	8	0.395	0.392	0.409±0.017	0.485	0.472	0.4835±0.011	0.521	0.5155	0.5115±0.004	0.506	0.5005	0.49675± 0.003	0.1307	0.0258	0.063
		0.529			0.612			0.701			0.663					
**Set 1**	11	0.583	0.556		0.678	0.645		0.604	0.6525		0.698	0.6805				
		0.536			0.673			0.698			0.678					
**Set 2**	11	0.558	0.547	0.5515±0.004	0.669	0.671	0.658±0.013	0.657	0.6775	0.665±0.012	0.71	0.694	0.68725± 0.006	0.02	0.0127	0.0028
		0.649			0.794			0.789			0.732					
**Set 1**	15	0.701	0.675		0.801	0.7975		0.768	0.7785		0.791	0.7615				
		0.689			0.768			0.81			0.836					
**Set 2**	15	0.674	0.6815	0.67825± 0.003	0.826	0.797	0.79725± 0.0002	0.826	0.818	0.79825± 0.019	0.798	0.817	0.78925± 0.027	0.0088	0.0082	0.015
		0.695			0.973			0.943			0.994					
**Set 1**	18	0.723	0.709		1.003	0.988		0.964	0.9535		0.892	0.943				
		0.736			0.954			1.056			1.106					
**Set 2**	18	0.785	0.7605	0.73475± 0.025	0.998	0.976	0.982±0.006	1.112	1.084	1.01875± 0.065	0.989	1.0475	0.99525± 0.052	<0.0001	<0.0001	<0.0001
		0.789			1.121			1.09			1.121					
**Set 1**	21	0.793	0.791		1.187	1.154		1.132	1.111		1.136	1.1285				
		0.81			1.168			1.21			1.231					
**Set 2**	21	0.823	0.8165	0.80375± 0.012	1.216	1.192	1.173±0.019	1.198	1.204	1.1575±0.046	1.25	1.2405	1.1845±0.056	<0.0001	<0.0001	<0.0001
		0.851			1.246			1.269			1.314					
**Set 1**	24	0.893	0.872		1.339	1.2925		1.204	1.2365		1.273	1.2935				
		0.865			1.321			1.325			1.336					
**Set 2**	24	0.925	0.895	0.8835±0.011	1.351	1.336	1.31425± 0.021	1.345	1.335	1.28575± 0.049	1.342	1.339	1.31625± 0.022	<0.0001	<0.0001	<0.0001

Supplementary Table-S2: Using TEM, the cell wall structure of WT and *dpb3*ΔΔ*dpb4*ΔΔ cells was observed and thickness of the cell wall was measured. The average of cell wall thickness of three different cells was considered. The statistically significant differences between the results of WT and mutant strains was analyzed using unpaired t test.

							**P value**
	**WT**	**Average**	**SD**	***dpb3ΔΔdpb4ΔΔ***	**Average**	**SD**	**WT Vs *dpb3ΔΔdpb4ΔΔ***
	78.99			208.05			
	90.57			156.55			
**TEM**	89.06	86.20667	5.140054	167.98	177.5267	22.08192	0.00469306

Supplementary Table-S3: Mean fluorescence intensity of aniline blue, concanavaline A, CFW stained cells of WT and *dpb3*ΔΔ*dpb4*ΔΔ strains was determined using flow cytometry. Data were represented as mean and Standard deviation from triplicate values. The statistically significant differences between the results of the WT and mutant was determined using one way ordinary ANOVA (Dunnett's multiple comparison test).

													P value		
	**WT**	**Average**	**SD**	***dpb3ΔΔ***	**Average**	**SD**	***dpb4ΔΔ***	**Average**	**SD**	***dpb3ΔΔdpb4ΔΔ***	**Average**	**SD**	**WT Vs *dpb3ΔΔ***	**WT Vs *dpb4ΔΔ***	**WT Vs *dpb3ΔΔdpb4ΔΔ***
**Aniline blue**	113			131.94			138			146.69					
	117			131.04			135			147.39					
	115	115	1.632993	130.96	131.3133	0.444322	137	136.6667	1.247219	147.27	147.1167	0.30565	0.0018	0.0006	0.0001
**Concanavaline A**	698			785			790			958					
	593			893			834			896					
	550	613.6667	62.16287	915	864.3333	56.81158	867	830.3333	31.54186	884	912.6667	32.4277	0.002	0.0049	0.0006
**CFW**	666			969			871			1088					
	689			1067			976			925					
	712	689	18.77942	987	1007.667	42.59369	1011	952.6667	59.48856	1055	1022.667	70.36255	0.0007	0.0024	0.0005

Supplementary Table-S4: The growth curve analyses of *dpb3*ΔΔ*dpb4*ΔΔ strain (**a**) without and with geldanamycin alone (107nM), fluconazole alone (6 μM), and a combination of both of the drugs, (**b**) without and with geldanamycin alone (107nM), amp B alone (10 nM), and a combination of both of the drugs, (**c**) without and with trichostatin A alone (0.8 μM), fluconazole alone (6 μM), and a combination of both of the drugs, and (**d**) without and with trichostatin A alone (0.8 μM), ampB alone (107nM), and a combination of both of the drugs. The absorbance measured at different time points and statistical analysis has been given as tables.

(a)

	Time(hrs)	*dpb3*ΔΔ *dpb4*ΔΔ	Average	Average of averages ± SD	*dpb3*ΔΔ *dpb4*ΔΔ+Geldanamycin	Average	Average of averages ± SD	*dpb3ΔΔ dpb4ΔΔ+ FLC*	Average	Average of averages ± SD	*dpb3ΔΔ dpb4ΔΔ+ FLC+Geldanamycin*	Average	Average of averages ± SD	P value<br>*dpb3ΔΔ dpb4ΔΔ+FLC vs dpb3ΔΔ dpb4ΔΔ+FLC+ Geldanamycin*
**Set 1**	0	0.094	0.0955	0.0995±0.004	0.094	0.0955	0.0995±0.004	0.098	0.0995	0.10375±0.004	0.098	0.0995	0.10275±0.003	>0.9999
		0.097			0.097			0.101			0.101			
**Set 2**	0	0.102	0.1035		0.103	0.1035		0.106	0.108		0.105	0.106		
		0.105			0.104			0.11			0.107			
**Set 1**	3	0.351	0.355	0.35175±0.003	0.358	0.3535	0.35425±0.007	0.318	0.322	0.3275±0.005	0.281	0.311	0.313±0.002	0.7981
		0.359			0.349			0.326			0.341			
**Set 2**	3	0.345	0.3485		0.345	0.355		0.321	0.333		0.265	0.315		
		0.352			0.365			0.345			0.365			
**Set 1**	6	1.277	1.2715	1.27775±0.006	1.254	1.2595	1.25975±0.0002	0.899	0.8825	0.875±0.007	0.762	0.7635	0.76225±0.001	<0.0001
		1.266			1.265			0.866			0.765			
**Set 2**	6	1.289	1.284		1.275	1.26		0.876	0.8675		0.751	0.761		
		1.279			1.245			0.859			0.771			
**Set 1**	9	2.349	2.4085	2.408±0.0005	2.412	2.383	2.4075±0.024	1.576	1.5845	1.58025±0.004	1.368	1.363	1.36325±0.0002	<0.0001
		2.468			2.354			1.593			1.358			
**Set 2**	9	2.339	2.4075		2.523	2.432		1.54	1.576		1.358	1.3635		
		2.476			2.341			1.612			1.369			
**Set 1**	12	2.692	2.6175	2.6155±0.002	2.654	2.633	2.64075±0.007	2.401	2.433	2.4465±0.013	1.475	1.503	1.50075±0.0022	<0.0001
		2.543			2.612			2.465			1.531			
**Set 2**	12	2.701	2.6135		2.665	2.6485		2.445	2.46		1.485	1.4985		
		2.526			2.632			2.475			1.512			
**Set 1**	15	2.742	2.6925	2.694±0.0015	2.778	2.7605	2.741±0.019	2.635	2.6165	2.63425±0.017	1.543	1.5775	1.568±0.0095	<0.0001
		2.643			2.743			2.598			1.612			
**Set 2**	15	2.754	2.6955		2.745	2.7215		2.692	2.652		1.521	1.5585		
		2.637			2.698			2.612			1.596			
**Set 1**	18	2.979	2.9165	2.9075±0.009	2.954	2.8945	2.859±0.035	2.739	2.78	2.78025±0.0002	1.731	1.7715	1.787±0.0155	<0.0001
		2.854			2.835			2.821			1.812			
**Set 2**	18	2.912	2.8985		2.812	2.8235		2.765	2.7805		1.795	1.8025		
		2.885			2.835			2.796			1.81			
**Set 1**	21	2.987	2.9775	2.97225±0.005	2.896	2.9325	2.9345±0.002	2.854	2.876	2.87475±0.001	2.052	2.032	2.027±0.005	<0.0001
		2.968			2.969			2.898			2.012			
**Set 2**	21	2.976	2.967		2.923	2.9365		2.835	2.8735		2.042	2.022		
		2.958			2.95			2.912			2.002			
**Set 1**	24	2.982	2.9835	2.97975±0.003	2.879	2.9315	2.94425±0.012	2.929	2.9305	2.9445±0.014	2.265	2.2395	2.29375±0.0542	<0.0001
		2.985			2.984			2.932			2.214			
**Set 2**	24	2.987	2.976		2.956	2.957		2.949	2.9585		2.375	2.348		
		2.965			2.958			2.968			2.321			

(b)

	Time(hrs)	*dpb3*ΔΔ *dpb4*ΔΔ	Average	Average of averages ± SD	*dpb3*ΔΔ *dpb4*ΔΔ+Geldanamyin	Average	Average of averages ± SD	*dpb3ΔΔ dpb4ΔΔ+* AmpB	Average	Average of averages ± SD	*dpb3ΔΔ dpb4ΔΔ+ Amp B+Geldanamycin*	Average	Average of averages ± SD	P value<br>*dpb3ΔΔ dpb4ΔΔ+* AmpB *vs dpb3ΔΔ dpb4ΔΔ+* AmpB *+Geldanamycin*
**Set 1**	0	0.103	0.104	0.10275±0.001	0.094	0.0955	0.0985±0.003	0.113	0.111	0.1085±0.002	0.108	0.1135	0.1085±0.005	0.9999
		0.105			0.097			0.109			0.119			
**Set 2**	0	0.103	0.1015		0.098	0.1015		0.105	0.106		0.103	0.1035		
		0.1			0.105			0.107			0.104			
**Set 1**	3	0.279	0.2865	0.29775±0.011	0.321	0.3095	0.323±0.013	0.156	0.1645	0.16775±0.003	0.179	0.172	0.172	0.9983
		0.294			0.298			0.173			0.165			
**Set 2**	3	0.321	0.309		0.332	0.3365		0.186	0.171		0.179	0.172		
		0.297			0.341			0.156			0.165			
**Set 1**	6	1.162	1.1535	1.154±0.0005	1.177	1.1665	1.1685±0.002	0.417	0.411	0.411	0.377	0.366	0.366	0.3693
		1.145			1.156			0.405			0.355			
**Set 2**	6	1.174	1.1545		1.169	1.1705		0.417	0.411		0.377	0.366		
		1.135			1.172			0.405			0.355			
**Set 1**	9	2.245	2.283	2.283	2.349	2.3085	2.30625±0.002	0.857	0.798	0.798	0.677	0.6655	0.6655	0.0007
		2.321			2.268			0.739			0.654			
**Set 2**	9	2.254	2.283		2.354	2.304		0.857	0.798		0.677	0.6655		
		2.312			2.254			0.739			0.654			
**Set 1**	12	2.554	2.576	2.576	2.592	2.5675	2.5695±0.002	1.713	1.622	1.622	1.387	1.3995	1.3995	<0.0001
		2.598			2.543			1.531			1.412			
**Set 2**	12	2.568	2.576		2.549	2.5715		1.713	1.622		1.387	1.3995		
		2.584			2.594			1.531			1.412			
**Set 1**	15	2.745	2.771	2.782±0.011	2.742	2.6925	2.70925±0.016	2.759	2.7575	2.7575	2.383	2.401	2.401	<0.0001
		2.797			2.643			2.756			2.419			
**Set 2**	15	2.784	2.793		2.754	2.726		2.759	2.7575		2.383	2.401		
		2.802			2.698			2.756			2.419			
**Set 1**	18	2.875	2.8935	2.91725±0.023	2.949	2.9015	2.95375±0.052	2.854	2.8595	2.8595	2.654	2.646	2.646	<0.0001
		2.912			2.854			2.865			2.638			
**Set 2**	18	2.898	2.941		2.958	3.006		2.854	2.8595		2.654	2.646		
		2.984			3.054			2.865			2.638			
**Set 1**	21	2.974	2.9695	3.0025±0.033	2.987	2.9775	3.0385±0.061	2.845	2.8785	2.928±0.049	2.832	2.8385	2.909±0.07	0.8818
		2.965			2.968			2.912			2.845			
**Set 2**	21	3.012	3.0355		3.098	3.0995		2.951	2.9775		2.985	2.9795		
		3.059			3.101			3.004			2.974			

(c)

														P value
	Time(hrs)	***dpb3*ΔΔ *dpb4*ΔΔ**	**Average**	**Average of averages ± SD**	***dpb3*ΔΔ *dpb4*ΔΔ+ Trichostatin A**	**Average**	**Average of averages ± SD**	***dpb3ΔΔdpb4 ΔΔ*+FLC**	**Average**	**Average of averages ± SD**	***dpb3ΔΔ dpb4ΔΔ+* FLC+Trichostatin A**	**Average**	**Average of averages ± SD**	***dpb3ΔΔdpb4 ΔΔ*+FLC *vs* *dpb3ΔΔdpb4 ΔΔ*+FLC+Trichostatin A**
		0.094			0.094			0.098			0.098			
**Set 1**	0	0.097	0.0955		0.097	0.0955		0.101	0.0995		0.101	0.0995		
		0.101			0.103			0.104			0.103			
**Set 2**	0	0.106	0.1035	0.0995±0.004	0.105	0.104	0.09975±0.004	0.106	0.105	0.10225±0.0027	0.107	0.105	0.10225±0.0027	>0.9999
		0.351			0.358			0.298			0.318			
**Set 1**	3	0.359	0.355		0.349	0.3535		0.312	0.305		0.352	0.335		
		0.326			0.346			0.305			0.298			
**Set 2**	3	0.365	0.3455	0.35025±0.004	0.374	0.36	0.35675±0.003	0.321	0.313	0.309±0.004	0.315	0.3065	0.32075±0.004	0.7994
		1.277			1.25			0.887			0.8			
**Set 1**	6	1.266	1.2715		1.255	1.2525		0.872	0.8795		0.849	0.8245		
		1.253			1.267			0.887			0.845			
**Set 2**	6	1.276	1.2645	1.268±0.003	1.259	1.263	1.25775±0.005	0.912	0.8995	0.8895±0.01	0.879	0.862	0.84325±0.01	0.0161
		2.349			2.442			1.572			1.046			
**Set 1**	9	2.468	2.4085		2.361	2.4015		1.589	1.5805		1.03	1.038		
		2.365			2.456			1.567			0.995			
**Set 2**	9	2.456	2.4105	2.4095±0.001	2.415	2.4355	2.4185±0.017	1.573	1.57	1.57525±0.005	1.025	1.01	1.024±0.005	<0.0001
		2.692			2.633			2.398			1.243			
**Set 1**	12	2.543	2.6175		2.646	2.6395		2.375	2.3865		1.305	1.274		
		2.569			2.622			2.354			1.23			
**Set 2**	12	2.612	2.5905	2.604±0.013	2.654	2.638	2.63875±0.007	2.385	2.3695	2.378±0.008	1.298	1.264	1.269±0.008	<0.0001
		2.742			2.756			2.621			1.413			
**Set 1**	15	2.783	2.7625		2.749	2.7525		2.612	2.6165		1.498	1.4555		
		2.698			2.747			2.594			1.435			
**Set 2**	15	2.721	2.7095	2.736±0.026	2.785	2.766	2.75925±0.006	2.635	2.6145	2.6155±0.001	1.479	1.457	1.45625±0.001	<0.0001
		2.979			2.971			2.723			1.543			
**Set 1**	18	2.854	2.9165		2.821	2.896		2.814	2.7685		1.598	1.5705		
		2.865			2.845			2.745			1.495			
**Set 2**	18	2.903	2.884	2.90025±0.016	2.869	2.857	2.8765±0.019	2.796	2.7705	2.7695±0.001	1.612	1.5535	1.562±0.001	<0.0001
		2.987			2.883			2.872			1.725			
**Set 1**	21	2.968	2.9775		2.959	2.921		2.895	2.8835		1.754	1.7395		
		2.956			2.798			2.869			1.684			
**Set 2**	21	2.978	2.967	2.97225±0.005	2.987	2.8925	2.90675±0.014	2.879	2.874	2.87875±0.004	1.736	1.71	1.72475±0.004	<0.0001
		2.982			2.983			2.934			1.845			
**Set 1**	24	2.985	2.9835		2.98	2.9815		2.941	2.9375		2.056	1.9505		
		2.998			2.896			2.975			1.864			
**Set 2**	24	2.958	2.978	2.98075±0.002	2.994	2.945	2.96325±0.018	2.986	2.9805	2.959±0.021	1.984	1.924	1.93725±0.021	<0.0001

(d)

														P value
	Time(hrs)	***dpb3*ΔΔ *dpb4*ΔΔ**	**Average**	**Average of averages ± SD**	***dpb3*ΔΔ *dpb4*ΔΔ+ Trichostatin A**	**Average**	**Average of averages ± SD**	***dpb3ΔΔ dpb4ΔΔ+* AmpB**	**Average**	**Average of averages ± SD**	***dpb3ΔΔ dpb4ΔΔ+* Amp B+Trichostatin A**	**Average**	**Average of averages ± SD**	***dpb3ΔΔdpb4 ΔΔ*+AmpB *vs* *dpb3ΔΔdpb4 ΔΔ*+AmpB+Trichostatin A**
		0.103			0.104			0.113			0.112			
**Set 1**	0	0.105	0.104		0.107	0.1055		0.109	0.111		0.107	0.1095		
		0.102			0.106			0.105			0.109			
**Set 2**	0	0.098	0.1	0.102±0.002	0.103	0.1045	0.105±0.0005	0.11	0.1075	0.10925±0.001	0.105	0.107	0.10825±0.001	0.9997
		0.279			0.311			0.186			0.213			
**Set 1**	3	0.294	0.2865		0.287	0.299		0.186	0.186		0.231	0.222		
		0.267			0.279			0.175			0.225			
**Set 2**	3	0.287	0.277	0.28175±0.0047	0.298	0.2885	0.29375±0.0052	0.198	0.1865	0.18625±0.0002	0.241	0.233	0.2275±0.005	0.021
		1.162			1.143			0.417			0.4			
**Set 1**	6	1.145	1.1535		1.161	1.152		0.405	0.411		0.418	0.409		
		1.156			1.136			0.456			0.412			
**Set 2**	6	1.167	1.1615	1.1575±0.004	1.172	1.154	1.153±0.001	0.421	0.4385	0.42475±0.013	0.454	0.433	0.421±0.012	0.9881
		2.245			2.319			0.857			0.657			
**Set 1**	9	2.321	2.283		2.351	2.335		0.739	0.798		0.677	0.667		
		2.227			2.359			0.816			0.631			
**Set 2**	9	2.412	2.3195	2.30125±0.0182	2.243	2.301	2.318±0.017	0.769	0.7925	0.79525±0.002	0.684	0.6575	0.66225±0.004	<0.0001
		2.554			2.585			1.713			1.385			
**Set 1**	12	2.598	2.576		2.661	2.623		1.531	1.622		1.384	1.3845		
		2.532			2.543			1.726			1.298			
**Set 2**	12	2.578	2.555	2.5655±0.010	2.678	2.6105	2.61675±0.006	1.612	1.669	1.6455±0.023	1.387	1.3425	1.3635±0.021	<0.0001
		2.745			2.839			2.759			2.401			
**Set 1**	15	2.797	2.771		2.787	2.813		2.756	2.7575		2.349	2.375		
		2.812			2.822			2.746			2.359			
**Set 2**	15	2.765	2.7885	2.779750.0087	2.795	2.8085	2.81075±0.002	2.739	2.7425	2.75±0.007	2.423	2.391	2.383±0.008	<0.0001
		2.875			2.889			2.854			2.654			
**Set 1**	18	2.912	2.8935		2.854	2.8715		2.865	2.8595		2.685	2.6695		
		2.865			2.872			2.895			2.662			
**Set 2**	18	2.889	2.877	2.88525±0.0082	2.885	2.8785	2.875±0.003	2.871	2.883	2.87125±0.011	2.671	2.6665	2.668±0.0015	<0.0001
		2.974			2.878			2.845			2.845			
**Set 1**	21	2.965	2.9695		2.884	2.881		2.912	2.8785		2.865	2.855		
		2.945			2.915			2.875			2.912			
**Set 2**	21	2.965	2.955	2.96225±0.0072	2.896	2.9055	2.89325±0.012	2.906	2.8905	2.8845±0.006	2.886	2.899	2.877±0.022	0.9179

Supplementary Table-S5: The growth curve analyses of WT, *dpb3*ΔΔ, *dpb4*ΔΔ, and *dpb3*ΔΔ*dpb4*ΔΔ strains were carried out with 5FC. The absorbance measured at different time points and statistical analysis has been given.

														P value		
	Time(hrs)	WT	Average	Average of averages ± SD	*dpb3*ΔΔ	Average	Average of averages ± SD	*dpb4*ΔΔ	Average	Average of averages ± SD	*dpb3*ΔΔ *dpb4*ΔΔ	Average	Average of averages ± SD	*WT vs dpb3ΔΔ*	*WT vs dpb4ΔΔ*	*WT vs dpb3ΔΔ dpb4ΔΔ*
**Set 1**	0	0.111	0.1095	0.1085±0.001	0.103	0.106	0.105±0.001	0.108	0.1035	0.10525±0.001	0.106	0.1035	0.1035	0.9994	0.9996	0.9985
		0.108			0.109			0.099			0.101					
**Set 2**	0	0.106	0.1075		0.103	0.104		0.106	0.107		0.103	0.1035				
		0.109			0.105			0.108			0.104					
**Set 1**	2	0.171	0.1715	0.1705±0.001	0.198	0.2035	0.20025±0.003	0.203	0.2045	0.21125±0.006	0.2	0.205	0.214±0.009	0.7917	0.6051	0.5576
		0.172			0.209			0.206			0.21					
**Set 2**	2	0.169	0.1695		0.205	0.197		0.215	0.218		0.231	0.223				
		0.17			0.189			0.221			0.215					
**Set 1**	4	0.376	0.371	0.369±0.002	0.398	0.404	0.41125±0.007	0.403	0.4095	0.4185±0.009	0.417	0.4145	0.42775±0.013	0.5791	0.4581	0.3238
		0.366			0.41			0.416			0.412					
**Set 2**	4	0.359	0.367		0.421	0.4185		0.424	0.4275		0.436	0.441				
		0.375			0.416			0.431			0.446					
**Set 1**	6	0.62	0.6305	0.63625±0.005	0.733	0.737	0.74925±0.012	0.743	0.741	0.7565±0.015	0.754	0.751	0.76475±0.013	0.0185	0.0116	0.0067
		0.641			0.741			0.739			0.748					
**Set 2**	6	0.609	0.642		0.754	0.7615		0.765	0.772		0.785	0.7785				
		0.675			0.769			0.779			0.772					
**Set 1**	8	1.108	1.1075	1.11425±0.006	1.231	1.243	1.25175±0.008	1.243	1.25	1.27125±0.021	1.265	1.2785	1.2885±0.01	0.0036	0.0009	0.0003
		1.107			1.255			1.257			1.292					
**Set 2**	8	1.119	1.121		1.256	1.2605		1.284	1.2925		1.285	1.2985				
		1.123			1.265			1.301			1.312					
**Set 1**	10	1.666	1.6945	1.66175±0.032	1.889	1.845	1.86325±0.018	1.823	1.848	1.89375±0.045	1.823	1.898	1.90525±0.007	<0.0001	<0.0001	<0.0001
		1.723			1.801			1.873			1.973					
**Set 2**	10	1.56	1.629		1.858	1.8815		1.934	1.9395		1.878	1.9125				
		1.698			1.905			1.945			1.947					
**Set 1**	12	2.089	2.0665	2.12075±0.054	2.234	2.2275	2.2845±0.057	2.249	2.254	2.29175±0.037	2.226	2.2355	2.29875±0.063	0.0006	0.0003	0.0002
		2.044			2.221			2.259			2.245					
**Set 2**	12	2.12	2.175		2.324	2.3415		2.361	2.3295		2.345	2.362				
		2.23			2.359			2.298			2.379					
**Set 1**	14	2.344	2.3625	2.3975±0.035	2.412	2.3665	2.40475±0.038	2.412	2.4035	2.44175±0.038	2.379	2.362	2.4260±0.064	0.9955	0.5448	0.811
		2.381			2.321			2.395			2.345					
**Set 2**	14	2.487	2.4325		2.408	2.443		2.523	2.48		2.487	2.49				
		2.378			2.478			2.437			2.493					
**Set 1**	16	2.412	2.405	2.44±0.035	2.432	2.4865	2.4855±0.001	2.512	2.522	2.542±0.02	2.462	2.454	2.4875±0.033	0.5237	0.0363	0.4905
		2.398			2.541			2.532			2.446					
**Set 2**	16	2.512	2.475		2.456	2.4845		2.557	2.562		2.511	2.521				
		2.438			2.513			2.567			2.531					
